# Macro and Micro Diversity of *Clostridium difficile* Isolates from Diverse Sources and Geographical Locations

**DOI:** 10.1371/journal.pone.0031559

**Published:** 2012-03-02

**Authors:** Richard A. Stabler, Lisa F. Dawson, Esmeralda Valiente, Michelle D. Cairns, Melissa J. Martin, Elizabeth H. Donahue, Thomas V. Riley, J. Glenn Songer, Ed J. Kuijper, Kate E. Dingle, Brendan W. Wren

**Affiliations:** 1 Department of Infectious and Tropical Diseases, London School of Hygiene and Tropical Medicine, London, United Kingdom; 2 Public Health Laboratory London, Health Protection Agency, Division of Infection, The Royal London Hospital, London, United Kingdom; 3 UCL Centre for Clinical Microbiology, University College London, Royal Free Campus, London, United Kingdom; 4 Microbiology and Immunology, University of Western Australia, Perth, Western Australia, Australia; 5 College of Veterinary Medicine, Iowa State University, Ames, Iowa, United States of America; 6 Department of Medical Microbiology, Leiden University Medical Center, Albinusdreef, Leiden, Netherlands; 7 Nuffield Department of Clinical Laboratory Sciences, Oxford University, John Radcliffe Hospital, Oxford, United Kingdom; University of Hyderabad, India

## Abstract

*Clostridium difficile* has emerged rapidly as the leading cause of antibiotic-associated diarrheal disease, with the temporal and geographical appearance of dominant PCR ribotypes such as 017, 027 and 078. Despite this continued threat, we have a poor understanding of how or why particular variants emerge and the sources of strains that dominate different human populations. We have undertaken a breadth genotyping study using multilocus sequence typing (MLST) analysis of 385 *C. difficile* strains from diverse sources by host (human, animal and food), geographical locations (North America, Europe and Australia) and PCR ribotypes. Results identified 18 novel sequence types (STs) and 3 new allele sequences and confirmed the presence of five distinct clonal lineages generally associated with outbreaks of *C. difficile* infection in humans. Strains of animal and food origin were found of both ST-1 and ST-11 that are frequently associated with human disease. An in depth MLST analysis of the evolutionary distant ST-11/PCR ribotype 078 clonal lineage revealed that ST-11 can be found in alternative but closely related PCR ribotypes and PCR ribotype 078 alleles contain mutations generating novel STs. PCR ribotype 027 and 017 lineages may consist of two divergent subclades. Furthermore evidence of microdiversity was present within the heterogeneous clade 1. This study helps to define the evolutionary origin of dominant *C. difficile* lineages and demonstrates that *C. difficile* is continuing to evolve in concert with human activity.

## Introduction

*Clostridium difficile* is a Gram-positive, spore-forming anaerobic bacterium that is responsible for a variety of gastrointestinal diseases in humans and animals, collectively referred to as *C. difficile*-infection (CDI) [1], [2]. The pathogen is frequently associated with antibiotic treatment and the severity of CDI ranges from mild antibiotic-associated diarrhea to the life-threatening pseudomembranous colitis [3], [4]. *C. difficile* was recognized as a pathogen only three decades ago [3], [5], and the reported incidence of CDI has increased significantly in the last decade as new variants emerge. *C. difficile* is evolving rapidly as exemplified by the recent global emergence of the PCR ribotype 027 (RT027) clonal lineage associated with increased mortality and morbidity [6]. These strains were variously identified by genotyping methods such as North American pulsotype 1 (NAP1) by pulse-field gel electrophoresis (PFGE), BI by restriction endonuclease assay (REA), 027 by PCR ribotyping and as sequence type 1 (ST-1) by MLST. Recently the incidence of the RT027 appears to be in decline [7], [8], with the simultaneous appearance of other clonal lineages such as RT078 [7], [8], [9], [10]. RT078, which is frequently isolated from livestock sources [11], has also been described as ‘hypervirulent’ and associated with infections as severe as those caused by RT027 strains [12], despite the fact that these lineages are distantly related [13]. Some PCR ribotypes can dominate in different geographical locations as exemplified by RT017 in Asia [14], [15] and Europe [16], [17].

Recent genetic epidemiological studies range from sequencing of selected conserved genes by multilocus sequence typing (MLST) to whole genome analysis [13], [18], [19], [20]. Lemee *et al.* used MLST to identify three divergent lineages, with A-B+ (toxin A negative, toxin B positive) isolates forming one of these lineages [20]. The derived STs lacked correlation with geographic source or extent of virulence [20], [21]. Comparative phylogeny using whole genome microarrays identified three apparent hypervirulent clonal lineages (RT017, RT027 and RT078) as well as a fourth heterogeneous grouping [19]. A whole genome sequencing (WGS) approach based on single nucleotide polymorphisms (SNPs) in conserved core genes confirmed the existence of four clonal lineages [13]. Phylogenetic analysis revealed that strains in the epidemic lineages (RT017, RT027 and RT078) evolved independently over a million year time scale [13]. A second MLST scheme subsequently confirmed a similar population structure, but noted an additional clonal lineage associated with RT023 isolates [18], [22]. The fifth lineage was missed by WGS as this was based on the same strains as used in the microarray study and did not include RT023 isolates. RT078 isolates are generally associated with livestock sources, for example in one study it was identified in 94% of bovine and 83% of swine isolates [23]. This may represent niche adaptation, but *C. difficile* has also been identified on salads [24] and vegetables [25], and RT078 was identified as a contaminant of grocery vegetables [26]. Although unproven, there is some evidence suggesting a link between livestock, food and human cases, and this, combined with the rise of community acquired CDI [27], has suggested that *C. difficile* may be a food borne pathogen [28].

Comparative phylogenomics, MLST and whole genome sequencing have indicated that the RT078 lineage is highly divergent from the other *C. difficile* lineages [13], [18], [19]. Dingle *et al* suggested that RT078 was a divergent non-toxigenic strain that gained the PaLoc (Pathogenicity Locus which includes the two major toxins; toxin A and toxin B) after niche adaptation. This was implied due to high sequence similarity of the RT078/ST-11 PaLoc region to other RTs, despite the high divergence in all the MLST alleles [22]. This evidence is further compounded by the considerable variability of the PaLoc sequence: there are 28 variants currently recognized [29], which can alter toxin properties and/or production including loss of production of toxin A (A-B+) or both toxins (A-B−) [30], [31]. This lineage appears to contain one RT represented by a single ST designated ST-11 [22]. However PCR ribotyping has shown that RT126 is closely related to RT078 as it differs by the loss of a single band on the amplified DNA banding pattern [32]. Interestingly RT126 has been identified in livestock, for example in 11% of neonate pigs [23]. Rupnik *et al* also reported that the RT078 PCR ribotyping banding patterns were similar to that of RT033, RT066 and RT045 [33]. In our previous study the human/animal HA2 clade which consisted predominantly of RT078 isolates, also contained a single RT126 isolate (JGS688) [19]. Recently Knetsch *et al* have shown, using hypervirulent lineage specific markers and PCR-ribotyping, that RT078 forms a phylogenetically coherent group with RT033, RT045, RT066, RT126 and RT127 [34].

To date, MLST studies on *C. difficile* have focused on human isolates from limited geographical regions. In this study we use MLST to investigate the population structure of 385 strains isolated from diverse human, animal and food sources, isolated from three continents and containing 67 PCR ribotypes. Analysis of our data, together with 80 previously published STs and 43 unpublished STs (from pubmlst.net) (123 STs in total) identified the five lineages associated with outbreaks of CDI in humans and demonstrated significant heterogeneity within the two clades which contain hypervirulent ST-11 (RT078) and ST-1 (RT027). These studies confirm the continued evolution of *C. difficile* hypervirulent lineages and the association of these strains with animal and potentially food sources.

## Materials and Methods

### Strains

**Figure 1:**
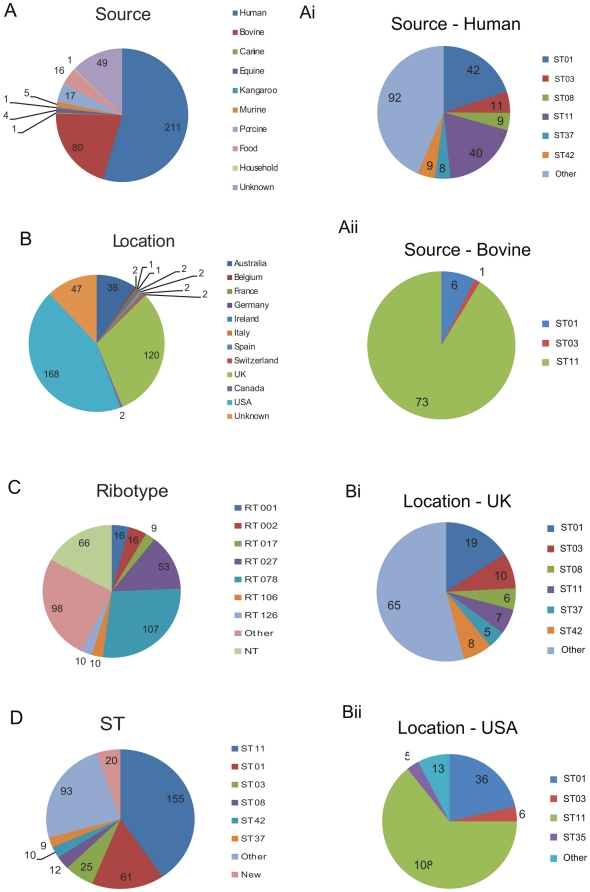
Breakdown of 385 *C. difficile* isolates in MLST study. A) Source of isolate (where given), Ai) breakdown by ST of human isolates (other = 57 additional STs), Aii) breakdown by ST of bovine isolates, B) Country of origin of isolate (where given), Bi) breakdown of UK isolates by ST (other = 47 additional STs), Bii) breakdown of USA isolates by ST, C) PCR ribotype (RT) of isolate (NT = RT not indicated and not tested, other = 61 additional RTs), D) Sequence type determined by this study (other = 42 additional STs).

The 385 strains investigated in this study were 211 human isolates (including 21 PCR ribotypes), 109 animal isolates (80 bovine, 17 porcine, 4 equine, 5 murine, 1 macropine [kangaroo], 1 canine), 16 food isolates, 1 household and 49 with unspecified source (figure 1, data S1). Geographically these were isolated in Europe (132), North America (170) and Australia (36) and 47 with unspecified location (figure 1). Within the 385 strains were 67 known PCR ribotypes (breadth component of study). The depth component of the study included 53 027/BI/NAP1 isolates and 107 RT078 both of which include clinical, animal and food isolates.

### MLST

*C. difficile* strains were sequence typed using the scheme described by Griffiths *et al* using seven housekeeping genes (*adk*, *atpA*, *dxr*, *glyA*, *recA*, *sodA* and *tpi*) [18]. Briefly, spores were incubated on Brazier's agar (Bioconnections, UK) for 24–72 h in an anaerobic environment at 37°C (Don Whitley Scientific). Three colonies were inoculated into 10 ml BHI, pre-equilibrated in an anaerobic environment. Genomic DNA was extracted either by phenol chloroform [35] or Tris-EDTA boilate [18]. High-throughput multilocus sequence typing was performed as per Griffiths *et al* [18]. Products of nucleotide sequencing reactions were analysed on an ABI 3730 (Applied Biosystems, USA) and nucleotide sequences extracted using Chromas v1.61 (Queensland, Australia). Allele designations were obtained by parsing forward and reverse sequencing reads through the *C. difficile* pubMLST batch profile query page (http://pubmlst.org/cdifficile/). Novel STs (18) and existing *C. difficile* pubMLST database (ST = 123; 80 previously published, 43 unpublished used with submitter's permission) were analysed using MAFFT (mafft.cbrc.jp) and Archeopteryx (www.phylosoft.org/archaeopteryx). Phylogenies were calculated by MAFFT and MrBayes [36], [37]. MAFFT used the neighbor-joining method [38] using all ungapped sites with 1000 boot strap resamplings. MrBayes used concatenated allele DNA sequences with a codon model, GTR+I+_ model (a General Time Reversible model with a proportion of invariable sites), heating of 0.5, 1,000,000 iterations with a 50% burn-in, 4 chains with the cold chain sampled ever 100 iterations and invgamma distribution. Phylogeny consensus and Bayesian probabilities (p) were calculated using the last 1501 recorded cold chain trees (iteration 850,000 to 1,000,000) from three independent runs.

### PCR ribotyping

PCR ribotyping was performed as described previously with some modifications [39]. Briefly, bacteria were harvested from 48 h anaerobic cultures on blood agar. Cells were resuspended into 5% (wt/vol) solution of Chelex-100 (Bio-Rad, United Kingdom) and heated to 100°C for 20 min. The suspension was separated by centrifugation (13000 g for 12 min) and the supernatant (5 µl) was added to a 45 µl-PCR mixture containing 25 µM of each primer: (CTGGGGTGAAGTCGTAACAAGG) and (GCGCCCTTTGTAGCTTGACC), 2.5 u HotStar Taq DNA polymerase (Qiagen, United Kingdom), 0.4 mM dNTPs and 3.75 mM MgCl_2_ per reaction. Reaction mixture was subjected to 30 cycles of 9°C for 1 min, 92°C for 1 min, 55°C for 1 min and 72°C for 1.5 min. This was followed by 95°C for 1 min, 55°C for 45 sec and 72°C for 5 min. The PCR products were concentrated to 20 µl by heating at 75°C for 40 mins. Electrophoresis was done at 100 mA in 3% precast 0.5% Tris-acetate-EDTA (TAE) agarose gels (BioRad, UK) containing ethidium bromide for 3.5 h. Banding patterns were analysed using GelCompar software (Applied Maths NV, Belgium). Dendrogram produced using GelCompar Software (Applied Maths, Belgium) using dice coefficient and UPGMA clustering with 1% tolerance and 0.5 optimization.

## Results

### Breadth study

The breadth study was designed to investigate the diversity of *C. difficile* isolates beyond previous studies by using diverse sources by host (human, animal and food), geographical location (North America, Europe and Australia) and PCR ribotype (figure 1, data S1). MLST analysis of 385 diverse *C. difficile* strains has identified 48 known STs, 18 novel STs and 3 new allele sequences. Previous studies investigated over 50 RTs and reported that there are 74 confirmed RT/ST associations (i.e. RT027 isolates are ST-1 and *vice versa*) [18], [22]. In this study we analysed strains from 67 PCR ribotypes (including 29 previously untyped by MLST) and found 86 previously described RT/ST associations (e.g. RT027/ST-1), 18 novel STs paired to 20 previously untyped RTs (1 ST was present in 3 RTs) (e.g. RT029/ST-137), 17 previously described STs with untyped RTs (e.g. RT042/ST-6) and 8 RTs had a new ST associated (e.g. RT268 was associated with ST-3, previously identified only in RT001, RT009, RT072, RT115 & RT262) (data S4).

**Figure 2:**
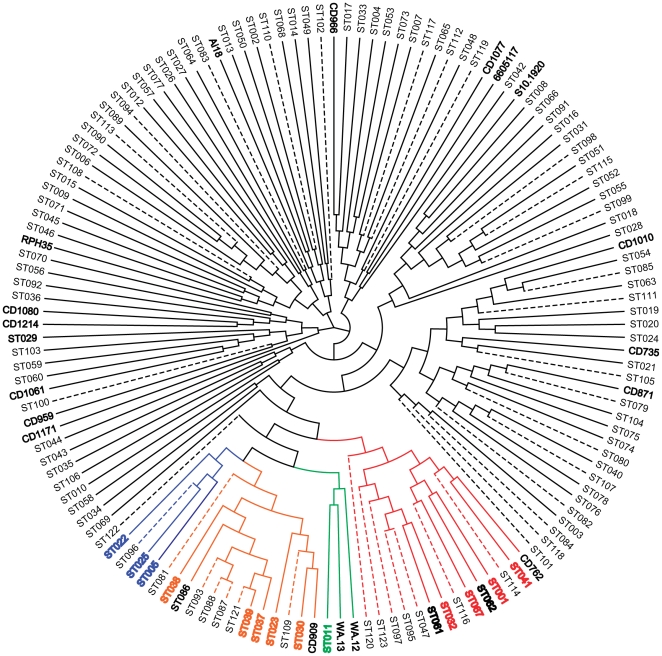
MAFFT alignment phylogeny of STs. MAFFT alignment phylogeny using neighbor-joining method [**38**] of *C. difficile* MLST database (ST = 123) plus 18 novel STs from this study. Leaf designations; STxxx = sequence type from MLST database, bold black = strain name representing novel ST from this study. Branch colouring; black = clade 1, red = clade 2 (inc ST-1/RT027), blue = clade 3 (inc ST-22/RT023), orange = clade 4 (inc ST-37/RT017), green = clade 5 (inc ST-11/RT078), dashed line = unpublished STs from pubMLST (used with permission). Leaf colouring; red = STs previously associated with clade 2, blue = STs previously associated with clade 3, brown = STs previously associated with clade 4, green = ST-11 [22].

**Figure 3:**
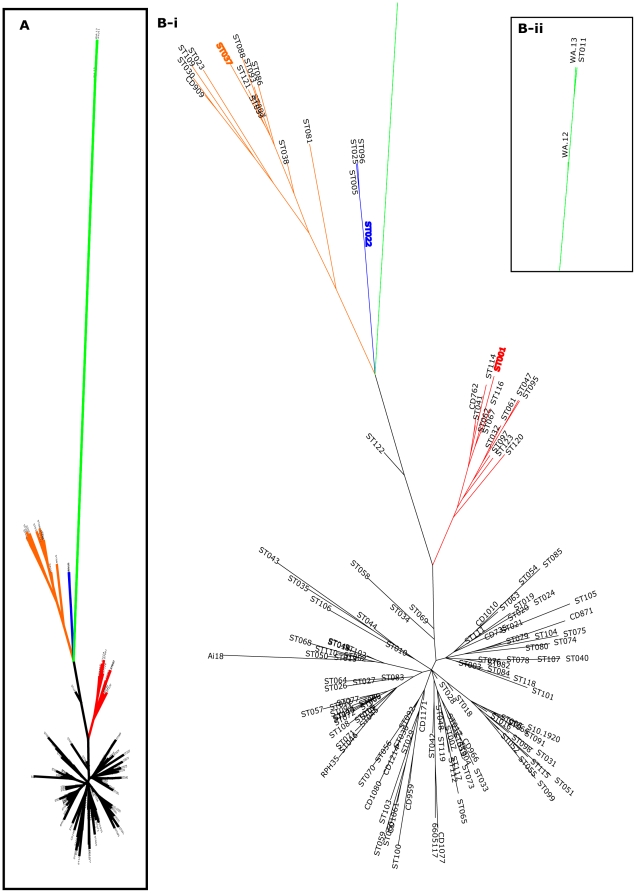
Relative evolutionary relatedness of five main subgroups and demonstration of microdiversity of subgroups. Branch colouring; black = clade 1, red = clade 2 (inc ST-1/RT027), blue = clade 3 (inc ST-22/RT023), orange = clade 4 (inc ST-37/RT017), green = clade 5 (inc ST-11/RT078). A) overview of phylogeny, B-i) detail of clades 1–4, B-ii) detail of clade 5.

**Figure 4:**
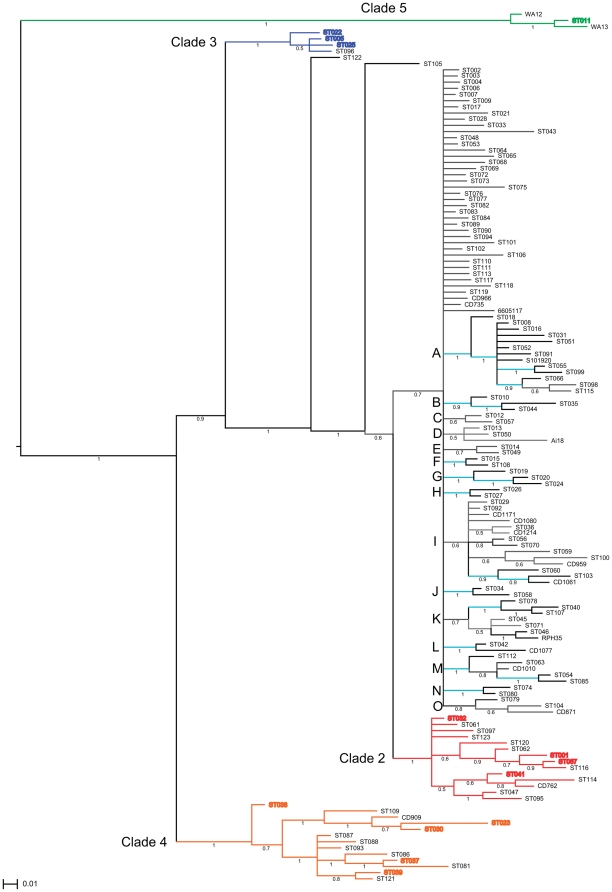
Microdiversity of clade 1 within the MLST phylogram. Branch colouring; black = clade 1 (light blue branch indicates microdiversity in clade 1 (p≥0.9)), red = clade 2 (inc ST-1/RT027), blue = clade 3 (inc ST-22/RT023), orange = clade 4 (inc ST-37/RT017), green = clade 5 (including ST-11/RT078). Leaf colouring; red = STs previously associated with clade 2, blue = STs previously associated with clade 3, brown = STs previously associated with clade 4, green = ST-11 [22].

Phylogenetic analysis using the MLST database (ST = 123, 01 Aug 2011) was comprised of 80 previously published, 43 newly described STs (used with the submitter's permission) plus the 18 novel ST from this study. The analysis concurred with Griffiths *et al* that there appeared to be five main clades (figure 2, 3, 4).

Clade 1 consists of 106 STs (figure 2), which are very heterogeneous in terms of alleles and RTs. No robust major subdivisions were detected in clade 1 by MAFFT analysis (supplementary figures S1, S2, S3, S4, S5, S6, S7). However, previously unreported micro-diversity was observed in 15 small clusters (figure 4 ‘A’ to ‘O’, data S5). These mini clusters showed a range of STs linked to RTs. A robust mini cluster of 13 STs (mini cluster A, p = 1 (unequivocal)) contained a variety of PCR ribotypes; with divergent STs however, no 2 STs differed by more than 5 SNPs across all seven alleles. Examples are two RT050 (ST-18 and ST-16) were present, which differ by only a single SNP in *glyA*. Additionally, two RT002's were present (ST-8 and a new ST from this study:ST-146 (strain S10.1920) which differed in a single SNP in *tpi*. Mini cluster K, although less stable (p = 0.7) contains all three STs associated with RT013 (ST-45, ST-71 & ST-78) and two STs associated with RT087, ST-145 (strain RPH35) and ST-46. ST-46 is not unique to RT087, it is also present in RT191 and RT230.

Mini cluster B (P = 0.9) contained ST-10 & ST-44 that are both RT015. RT015 has increased in the UK from 50 isolates in 2007/8 to 330 isolates in 2009/10 and had the second highest associated mortality rate, after RT027 [7]. Mini cluster I contained both STs linked to RT21 (ST-56 & ST-70) and 2/3 RT011 STs (ST-36 and ST-138 (strain CD1214). Although the majority (11/15) of mini clusters contained three or fewer STs, four contained conserved RTs e.g. mini cluster G contains both STs associated with RT202, in mini cluster E both STs were associated with RT014, mini cluster D STs were also linked with RT014 (although ST-13 has been present in RT129 isolates). The two RT014 mini clusters (D & E) were not that stable within the phylogram (p = 0.5 and 0.7 respectively) and may be a single mini cluster of RT014 isolates. The consensus of these two mini clusters differs by only 1 SNP in *dxr* (between *dxr06* and *dxr02*) and 1 SNP in *tpi* (between *tpi01* and *tpi03*).

Interestingly strain AI18 (mini cluster D) looks to be a ST-13 that has acquired an *adk05* allele, which has previously only been associated with ST-11/RT078 isolates. This may represent homologous recombination rather than the acquisition of the 5 specific SNPs present in *adk05* (figure S1).

Clade 2 as defined by Dingle *et al.*, apart from ST-1, also contained ST-67, ST-32 and ST-41 [22]. We identified 5 isolates from these STs; two RT262 were ST-67, one untyped isolate was ST-32 and two isolates were ST-41 (one of which was a RT135). Clade 2 in this study consists of 14 STs (figure 2 & 4). Clade 2 may be subdividable into two subclades; a ST-1 subclade (2i) and a ST-32 subclade (2ii) (p = 0.83) (figure 2). Subclade 2i contained ST-1 (RT027), ST-67 (RT019 [18]), ST-41 (RT106/164/321 [22]), ST-62 (RT036) and ST-140 (strain CD762, RT111). Subclade 2ii contained 6 STs (ST-32, ST47, ST-61, ST-95, ST-97 & ST-123) with only ST-32 having a known RT (RT153 [18]). However in the Baysian cluster (figure 4) this was less robust; for example the ST-41, ST-114 and CD762 (ST-140) group (2i) cluster closer to ST-47 and ST-95 (2ii) but with Baysian probability values (p) or 0.6 and 0.5 shows that this part of the phylogram was not fully resolved.

Clade 3, includes ST-22/RT023, with 3 additional STs; ST-5 and ST-25 as previously reported [18], [22] plus ST-96 which has not been reported before in this clade (figure 2 & 4).

Clade 4 includes ST-37/RT017 and an additional 12 STs (figure 2 & 4). Clade 4 can be subdivided into two subclades (p = 1), an ‘ST-37’ subclade (4i) and an ‘ST-23’ subclade (4ii) (table 1). Subclade 4i contains 4 STs, including both RT017 STs (ST-37 & ST-86) as well as RT085 (ST-39). Subclade 4ii contains 8 STs including ST-142 (strain CD909, RT264) and ST-23 (RT138 [22]). Subclade 4ii has significant divergence from subclade 4i and may represent a sixth lineage. The formation of the subclades is mainly due to divergence in the *dxr*, *sodA* and *tpi* alleles (table 1). Interestingly, while alleles from clade 4 cluster (figure S1, S2, S3, S4, S5, S6, S7) for ST-23, *glyA14* represents a significant change within *glyA* phylogeny (figure S4) from *glyA08*, which suggests a recombination event.

**Table 1:** Sub-division of clade 4.

	RT	ST	*adk*	SNP	*atpA*	SNP	*dxr*	SNP	*glyA*	SNP	*recA*	SNP	*sodA*	SNP	*tpi*	SNP
	RT060	**38**	3		7		10	1	8		6		2		9	1
4i		**109**	3		12	1	10	1	18	1	6		18	3	15	3
	RT264	**CD909**	8	1	7		14	2	8		6		25	1	15	3
	RT138	**23**	3		7		14	2	14	3	11	1	16	2	15	3
		**30**	8	1	7		14	2	8		6		16	2	15	3
4ii		**87**	3		7		3		8		6		20	1	10	
		**88**	9	1	7		3		8		6		18	3	10	
		**93**	3		7		3		8		11	1	20	1	10	
	RT017	**86**	3		7		3		8		6		19	3	11	1
	RT017	**37**	3		7		3		8		6		9	1	11	1
		**81**	3		1	4	3		8		6		9	1	11	1
	RT085	**39**	3		7		10	1	8		7	1	2		10	
		**121**	3		7		3		8		7	1	2		10	
Con			3		7		3		8		6		2		10	

RT = PCR ribotype, ST = MLST sequence type, Con = most prevalent MLST alleles for clade 4, SNP = number of single nucleotide changes from consensus.

Clade 5 including ST-11/RT078 and an additional two STs; two novel STs as well as ST-11 (discussed below).

### Animal and food isolates

Sixty-three isolates taken from North American calves between 26/09/2006 and 15/10/2007 were MLST typed and the majority (46/63) were PCR ribotyped. Of these 57/63 were ST-11 and confirmed RT078, five isolates were ST-1 and confirmed as RT027 and one isolate was ST-3 (data S1). The single canine isolate was ST-2/RT020. The four equine isolates were ST-1/RT027, ST-03, ST-08/RT002 and ST-11/RT176. All five murine isolates were ST-35/RT002 but were all from the USA. The porcine isolates were predominantly (13/17) ST-11, the USA ST-11 porcine isolates were RT-126 (2) or RT-078 (3), whereas the Australian ST-11 were RT-237 (3). In addition, two USA porcine isolates were ST-048/RT002, whereas the Australian porcine isolate was ST-08/RT014 (AI18), as AI18 had an unusual *adk* allele. The kangaroo isolate was an ST-11/RT126. Interestingly the two Italian ST-11 clinical isolates were RT078, whereas the two Spanish ST-11 clinical isolates were RT126. All 16 food and 1 household isolates were from the USA and were ST-01 (7), ST-11 (8) or ST-61 (2). One ST-11 was a RT-176 but of those typed they were the usual ST-01/RT027 or ST-11/RT078.

### Identifying ancestral type

To investigate the ancestral root of *C. difficile*, MLST allele equivalent sequences were extracted from *C. botulinum* A BoNT/A1 (ATCC 19397) and *C. strichlandii* DSM 519 genome sequences. Based upon MAFFT analysis using the *C. difficile* MLST database, new STs from this study and the concatenated *C. botulinum* and *C. strichlandii* alleles were used to generate a phylogeny (figure S8). The phylogram using distance demonstrated that these are distantly related to *C. difficile*, but interestingly these are rooted closest to the ST-11 clade (figure S8).

### Depth study

To establish a correlation between MLST and the three frequently used typing methods of PCR-ribotyping, PFGE and REA, 53 isolates typed as RT027, NAP1 or BI were examined (and presumed to be RT027) (data S2). To reduce sampling bias the isolates examined were from diverse sources including human clinical cases of CDI, animals (bovine, equine) and food isolates from the US, UK, France and Canada (data S2). All 53 RT027 isolates were confirmed as ST-1. Analysis of 11 REA BI subtypes shows that ‘historic pre-2001’ isolates (BI-1 [1988], BI-2 [1991] and BI-5 [1995]) have the same ST as later epidemic RT027 (e.g. BI-6 [2003], BI-10 [2003] and BI-15 [2004]). This demonstrates that point mutations which affect the REA restriction sites within the genome are being acquired faster than in the seven housekeeping genes used for MLST sequences. BI-9, which was previously believed to be RT027 but did not cluster in the RT027 lineage [19] and subsequently confirmed as RT001 [13] and was shown to be ST-3. RT001 isolates have been shown to be ST-3 previously [18], [22].

In addition two RT176 isolates were also found to be ST-1.

**Figure 5:**
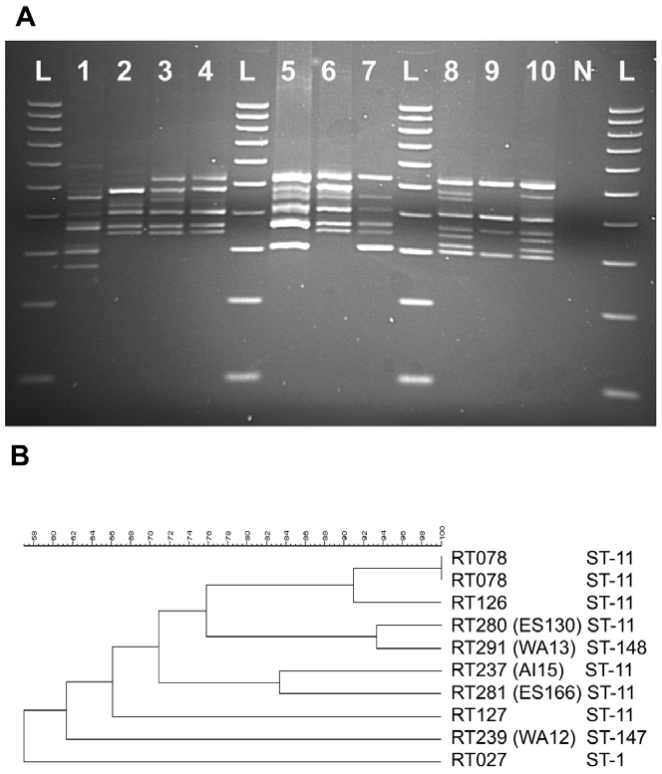
Clade 5 (inc ST-11) linked PCR ribotypes. A) PCR ribotype amplicon banding patterns as observed by agarose gel electrophoresis. Lane 1 - RT027 [ST-1], lane 2 - RT127 [ST-11], lane 3 - RT078 [ST-11], lane 4 - RT126 [ST-11], lane 5 - RT237 (AI15) [ST-11], lane 6 - RT078 [ST-11], lane 7 - RT281 (ES166) [ST-11], lane 8 - RT280 (ES130) [ST-11], lane 9 - RT239 (WA12) [ST-147], lane 10 - RT291 (WA13) [ST-148], N - negative control, L - Ladder. B) UPGMA dendrogram. Dendogram showing similarity of ST-11 associated ribotypes, rooted to RT027. Dendogram produced using GelCompar Software (Applied Maths, Belgium) using dice coefficient and UPGMA clustering with 1% tolerance and 0.5 optimization.

155 isolates were identified as ST-11 including all 106 previously designated RT078 (data S3). Thirty-one untyped animal and food sourced isolates were also found to be ST-11. ST-11 was also the only ST associated with RT126 (10/10 isolates), RT127 (3/3), RT237 (4/4), RT033 (1), RT280 (1) and RT281 (1) (figure 5, data S3). Although alleles can be present in more than one ST significantly all seven alleles associated with ST-11 are unique to this ST. This again reflects the distant relatedness of RT078 to the other known PCR ribotypes. Interestingly two Australian isolates (WA12 and WA13, ST-147 and ST-148 respectively) shared 6/7 alleles with ST-11 and a novel seventh allele (designated *adk10* and *tpi20* respectively). The *adk05* allele (ST-11) differs from the *adk* consensus by 5 unique SNPs, *adk10* (WA12) has only 2 of these SNPs (277 t→c, 483 a→g) suggesting either that *adk10* is an intermediary between consensus and *adk05* or that 3 SNPs have reverted back to consensus (figure S1). The *tpi20* (WA13) allele differs from the expected ST-11 *tpi* (*tpi08*) by 2 SNPs (198 t→c, 255 t→c), the former is also found only in unrelated *tpi06* and the latter appears to be a reversion of 255 c→t present only in *tpi08* and the related *tpi07* (figure S7). WA12 was identified as a RT239, A-B− cdt+ isolate and was originally from an unusual case of human bacteremia [40]. WA13 was typed as a RT291 and toxin A negative, toxin B positive (A-B+ toxinotype XXX). In terms of PCR ribotyping banding patterns, RT239 and RT291 are not considered similar to RT078 (figure 5B). WA13 (RT291) amplicon banding pattern was >92% similar to RT280 which was confirmed to be ST-11 (figure 5B). WA12 (RT239) amplicon banding pattern was <62% similar to any ST-11 PCR ribotype amplicon banding pattern (figure 5B). Recently Solomon *et al.* showed that RT078 isolates taken from Irish hospitals were very diverse (>40% similar) when analysed by rep-PCR [41]. Interestingly 2/4 RT237 and the single isolates of RT280 and RT281 were shown to be toxin A- and toxin B+ isolates, but were ST-11 by MLST.

Interestingly AI15 (RT237) came from the same pig farm as AI35 (RT237) but was isolated approximately 5 years earlier. At that time RT237 was one of a number of different ribotypes found but it now dominates in the same way as RT078 does elsewhere.

## Discussion

*C. difficile* is a diverse pathogen whose evolution has been driven by human activity with the emergence of clonal lineages such as RTs 017, 027 and 078. To date, MLST studies on *C. difficile* have focused on human isolates from limited geographical regions. In this study we have investigated the MLST profiles of 385 *C. difficile* isolates, including previously undocumented RTs, taken from clinical, animal and food isolates from geographically diverse locations. Despite the diverse origins of the isolates, the five subgroups previously reported were maintained, although further microdiversity within the subgroups was observed. By combining all available MLST data, there is evidence that clade 2 (including ST-1/RT027) and clade 4 (including ST-37/RT017) may consist of two divergent subclades. It is tempting to suggest that these virulent lineages may be evolving rapidly and diverging into potentially new virulent clones. Within clade 1 there was evidence of mini clusters which demonstrated some conservation of RT and identified the change in ST was the result of single SNPs within a single allele. Clustering of RTs in mini clusters demonstrated that these RTs had a recent common ancestor and, like the RT027 and RT078 lineages, are demonstrating signs of genome flux and potentially adaptation. RTs with multiple STs and *vice versa* demonstrate that the *C. difficile* genomes are constantly diverging and that this is not equally distributed through the genome. Indeed recently Castillo-Ramirez *et al* demonstrated that synonymous SNPs are mainly acquired through recombination rather than *de novo* mutations and emerging strains tend to have more non-synonymous SNPs that have not yet been purified by selection over time [42]. Therefore, the next ‘hypervirulent’ strain could potentially emerge from any lineage. For example, RT027 emerged as a major problem in North America around 2003 and in the UK in 2006 yet this clone was first identified in 1985 in France (CD196) [43]. Genome sequencing has revealed that CD196 and subsequent outbreak strains are genetically very similar and are identical by MLST. The same is true for RT023 and RT078, indeed RT023 was originally identified by PCR-ribotyping prior to 1985, but is now emerging as a problematic clone [7]. Conversely, RTs that have persisted within hospitals, for example RT001 and RT106 in the UK, and not considered ‘hypervirulent’ belong to the heterogenous clade 1.

Outbreak strains are often referred to as PCR ribotype 027/NAP1/BI, implying a strong correlation between all three typing methods. Recently Tenover *et al* showed that these typing methods do not always correlate. For example, only 84/92 RT027 isolates were also type-able as NAP1 and BI [44]. All three typing methods rely on the examination of DNA migration through a matrix, usually an agarose gel and comparison to a standard. Simple changes in DNA can have distinct changes in this pattern. For example, change in repeat copy number, interchromosomal homologous recombination for PCR-ribotyping [45] and changes in primer binding sites or SNPs within restriction sites for PFGE or REA can produce differences in banding patterns, which may result in the strain being designated as a different type or variant. Type BI determined by REA has at least 17 subtypes. Analysis by Griffith *et al* (2010) of 17 clinical RT027 identified one unique ST that was allocated ST-1. ST-1 was also found in a single RT036 isolate, a RT banding profile which differed by only a single band from RT027 [18]. We have confirmed that RT027, NAP1 or BI strains were all ST-1, with the exception of BI-9, (RT001,) which was reflected in the ST data (ST-3). Interestingly RT176, closely related to RT027 by MLVA, was also demonstrated to be ST-1, as were an additional 9 RTs, including RT036, RT111, RT135, RT153, RT176 and RT262 with RT027. Recently Nyc *et al* have shown that in Poland RT027 was found in low numbers, but RT176 was responsible for a recent outbreak [46]. The authors compared RT176 MLVA with a previous MLVA of European RT027 isolates [47] and demonstrated a high degree of similarity between these two PCR ribotypes. Also Knetsch have shown that a RT027 genetic marker was also found in RT176, as well as RT016, RT019, RT036, RT075, RT111, RT122, RT153, RT156, RT208 and RT273 [34]. Valiente *et al* also recently demonstrated that some of the strains typed as BI by REA and presumed to be RT027 were in fact RT176 and other RTs including RT198 and RT244 due to slight variation in banding patterns compared to the RT027 strains [48]. This demonstrates that MLST has lower discriminatory power than REA, ribotyping or pulse field typing, which could indicate strain divergence. Given that PCR-ribotyping is the most widely used typing method, MLST studies that confirm that the close grouping of different RTs such as RT176, RT198 and RT244 grouping with the notorious RT027, suggests that there should be heightened awareness of these closely related RTs.

RT078 has recently emerged as a clinical problem after a long documented association with livestock (reviewed in [49]). Interestingly, microarray, MLST and whole genome sequencing studies have shown this RT to be highly divergent from other known RTs and has been suggested that the last common ancestor was 1.1–6.4 million years ago [13]. Dingle *et al* suggested this maybe a non-toxigenic strain that has recently acquired the PaLoc due to sequence similarity to clade 3 (RT023) PaLoc [22]. Our studies have shown that ST-11 is ubiquitous and is found in Australia, the USA and Europe in clinical, animal and food sources. Interestingly, six additional RTs were in ST-11, demonstrating that divergence within the 16S-23S PCR ribotyping region have not yet been reflected as divergence in the MLST alleles, suggesting that the emergence of these RT's are recent events. However local variation in Australia has resulted in novel alleles and RTs. There are also potential geographic differences observed in this clade; porcine ST-11 isolates differed in RT pattern between USA (RT078/RT126) and Australia (RT237). However, these differences are not reflected in the wider population; for example, an ‘American’ ST-11/RT126 was isolated from an Australian native Kangaroo and an Australian clinical isolate. This may be attributed to the limited number of animal isolates. Interestingly the novel *adk* allele (*adk10*) appears to be between ST-11 and all other *adk* alleles (supplementary figure S1). Three clustered SNPs differentiate *adk10* from *adk05* (bp 463, 469 & 471). The *adk* allele can give us an insight into the evolution of the ST-11 because of one of three possibilities. 1) *adk10* is ancestral to the ST-11 allele (*adk05*), 2) *adk05* has undergone reversions towards the consensus to generate *adk10* or *3*) recombination between ST-11 and an unknown ST(s) has resulted in a mosaic allele.

Evolution of ST-11 can also be observed using the *tpi* allele. In the case of *tpi20* the deviation from the expected *tpi8* involves 2 SNPs, one to the ancestral state and a second that mirrors a SNP only found in ST-6. For this to be a mosaic, two independent recombination events must occur or one from a currently unknown allele. It is interesting to note that the RT078 clone has spread globally with the ST remaining stable except in Australia where it appears to be acquiring SNPs. This is perhaps due to RT078 becoming more prevalent in humans than animals and is reverting to a genotype more applicable to this host and as such appears to be reverting to the consensus derived from mainly human isolates.

Interestingly, when *C. botulinum* and *C. strichlandii* were added to the clustering they rooted more closely to the ST-11 clade, which may suggest that these represent an older lineage, having been associated with animals prior to the emergence of *Homo sapiens*. However, He *et al.*, using SNP analysis on the whole genome of *C. difficile*, alongside *C. bartletti* and *C. hiranonis* identified the root to among the other four clades [13]. *C. botulinum* and *C. strichlandii* were selected over *C. bartletti* and *C. hiranonis* due to the lack of useable sequence equivalent to the *C. difficile* alleles. For example *C. bartletti* and *C. hiranonis* lack *sodA* and *C. hiranonis* lacks *glyA* and *C. bartletti* has only a partial BLAST match (141/210 bp identities) to the 516 bp allele. The different data explain the different rooting and it is arguable whether highly conserved sequence (for example 16S) are more suitable than whole genomes which have greater variability and horizontal transfer to estimate ancestral type.

Interestingly one of the novel STs (ST-148) that was a variant on ST-11 was shown to be a toxin A negative, toxin B positive toxin variant, previously only found in RT017 isolates. Recently it has been shown that novel non-RT017 A-B+ isolates are present in Australia and Asia [29], [50]. Rupnik *et al.* have shown that the loss of toxin A expression is not necessarily due to loss of all or part of *tcdA*, but rather due to a nonsense point mutation that abrogate expression [51]. This suggests that, rather than RT017 A-B+ isolates diverging into novel RTs and/or genotypes, it is possible that *C. difficile* may spontaneously lose the ability to express TcdA. The second ST-11 variant was A-B− (RT239, ST-147), which previous microarray data suggested was through spontaneous loss of the PaLoc as this occurred in different lineages [19].

With the current PCR ribotyping database containing at least 377 known PCR ribotypes (Vall Hall, personal communication 26/10/2011), population studies have been limited by the breadth of the samples tested. The continued collection and typing of diverse *C. difficile* strains is vital for monitoring and emergence (and disappearance) of evolving virulent and hypervirulent clones. The MLST analysis performed on diverse strains from different geographic locations and environments has revealed new links between closely related RTs and STs, which has provided an insight into the microevolution of *C. difficile*. Our analysis of the potentially divergent clonal linages has broadened our understanding the evolution of this enigmatic pathogen.

## Supporting Information

Figure S1 **MAFFT circular phylogram with** ***adk*** **alleles.** A) Circle phylogeny coloured by allele. B) MrBayes SNP phylogram of *adk* alleles coloured by allele. SNPs indicated on branch (e.g. 22a→g (8V→I) indicates a SNP that changes the 22^nd^ base from adenine to guanine, brackets indicate a non-synonymous change ie a valine (V) to an isoleucine (I)).
(PDF)

Figure S2 **MAFFT circular phylogram with** ***atp*** **alleles.** A) Circle phylogeny coloured by allele. B) MrBayes SNP phylogram of *atp* alleles coloured by allele. SNPs indicated on branch. * indicates a non-unique SNP that occurs in more than one place on phylogram.
(PDF)

Figure S3 **MAFFT circular phylogram with** ***dxr*** **alleles.** A) Circle phylogeny coloured by allele. B) MrBayes SNP phylogram of *dxr* alleles coloured by allele. SNPs indicated on branch. * indicates a non-unique SNP that occurs in more than one place on phylogram.
(PDF)

Figure S4 **MAFFT circular phylogram with** ***glyA*** **alleles.** A) Circle phylogeny coloured by allele. B) MrBayes SNP phylogram of *glyA* alleles coloured by allele. SNPs indicated on branch. * indicates a non-unique SNP that occurs in more than one place on phylogram. ! indicates a SNPs in *glyA04* which has reverted to ancestral in *glyA03* and into a non-synonymous SNP in *glyA14*.
(PDF)

Fgure S5 **MAFFT circular phylogram with** ***recA*** **alleles.** A) Circle phylogeny coloured by allele. B) MrBayes SNP phylogram of *recA* alleles coloured by allele. SNPs indicated on branch. * indicates a non-unique SNP that occurs in more than one place on phylogram.
(PDF)

Figure S6 **MAFFT circular phylogram with** ***sodA*** **alleles.** A) Circle phylogeny coloured by allele. B) MrBayes SNP phylogram of *sodA* alleles coloured by allele. SNPs indicated on branch. * indicates a non-unique SNP that occurs in more than one place on phylogram.
(PDF)

Figure S7 **MAFFT circular phylogram with** ***tpi*** **alleles.** A) Circle phylogeny coloured by allele. B) MrBayes SNP phylogram of *tpi* alleles coloured by allele. SNPs indicated on branch. * indicates a non-unique SNP that occurs in more than one place on phylogram.
(PDF)

Figure S8 **MAFFT Rooted phylogram.** A) Scaled phylogram displaying relationship of *C. botulinum* A BoNT/A1 (ATCC 19397) and *C. strichlandii* DSM 519 to *C. difficile*, B) MAFFT unscaled phylogram showing location of *C. botulinum* A BoNT/A1 (ATCC 19397) and *C. strichlandii* DSM 519 within *C. difficile* population. Black branches = clade 1, orange branches = clade 2 (inc ST-1), green ranches = clade 3 (inc ST-23) blue branches = clade 4 (inc ST-37), red branches = clade 5 (inc ST-11).
(PDF)

Data S1 **Strains in study (inc MLST data).**
(PDF)

Data S2 **Strains in PCR ribotype 027 depth study.**
(PDF)

Data S3 **Strains in PCR ribotype 078 depth study.**
(PDF)

Data S4 **PCR ribotypes associated with RT. Griffiths = data taken from Griffiths** ***et al*** **, Dingle = data taken from Dingle** ***et al*** **, Stabler = data from this study, PCR ribotypes (RT) tested (highlighted indicates previously untested RT), Nos = number of isolates with RT and Sequence Type (ST) combination in this study, ST = sequence type (highlight indicates a new RT/ST association, ‘new’ indicates a novel ST.**
(PDF)

Data S5 **Mini clusters within clade 1.** Closely related sequence types cluster in to mini clades within the larger heterogenous clade 1. MC = micro cluster identifier, *adk*, *atpA*, *dxr*, *glyA*, *recA*, *sodA* and *tpi* columns indicate allele numbers (red = consensus, ‘.’ = same as consensus), SNP column indicates the number of single nucleotide polymorphisms between consensus allele and deviant allele (1Δ indicates a single base pair deletion within *sodA* allele 21), associated RTs column indicates any known PCR ribotypes associated with this ST.
(PDF)
