# Supplementary figures and images for: Macro and Micro Diversity of Clostridium difficile Isolates from Diverse Sources and Geographical Locations
Source: PLoS One. 2012 Mar 2;7(3):e31559. doi: 10.1371/journal.pone.0031559 (PMC3292544; doi:10.1371/journal.pone.0031559)

1 = Red  
2 = Green  
3 = Blue  
4 = Yellow

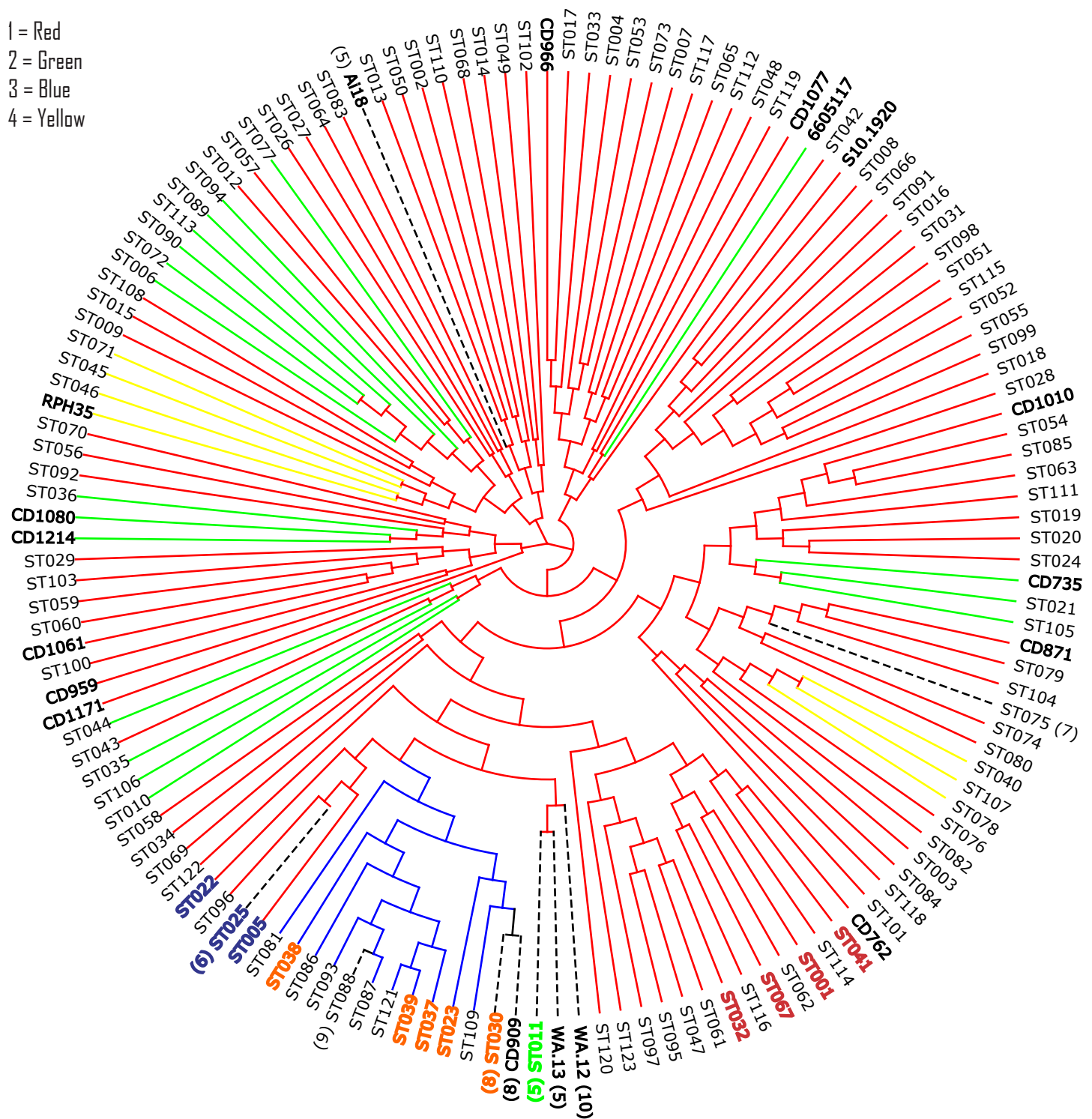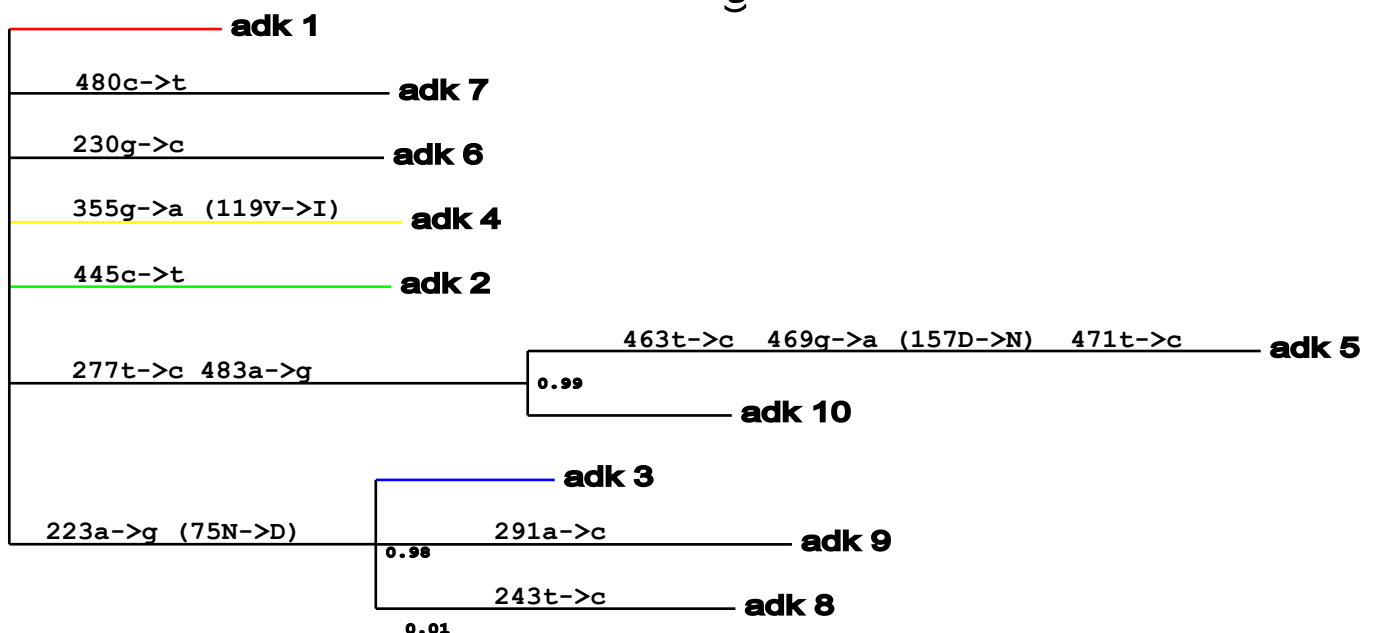

Supplement: Figure S1 — MAFFT circular phylogram with adk alleles. A) Circle phylogeny coloured by allele. B) MrBayes SNP phylogram of adk alleles coloured by allele. SNPs indicated on branch (e.g. 22a→g (8V→I) indicates a SNP that changes the 22nd base from adenine to guanine, brackets indicate a non-synonymous change ie a valine (V) to an isoleucine (I)). (PDF) [file pone.0031559.s001.pdf]

1 = Red  
5 = Green  
7 = Blue  
6 = Yellow

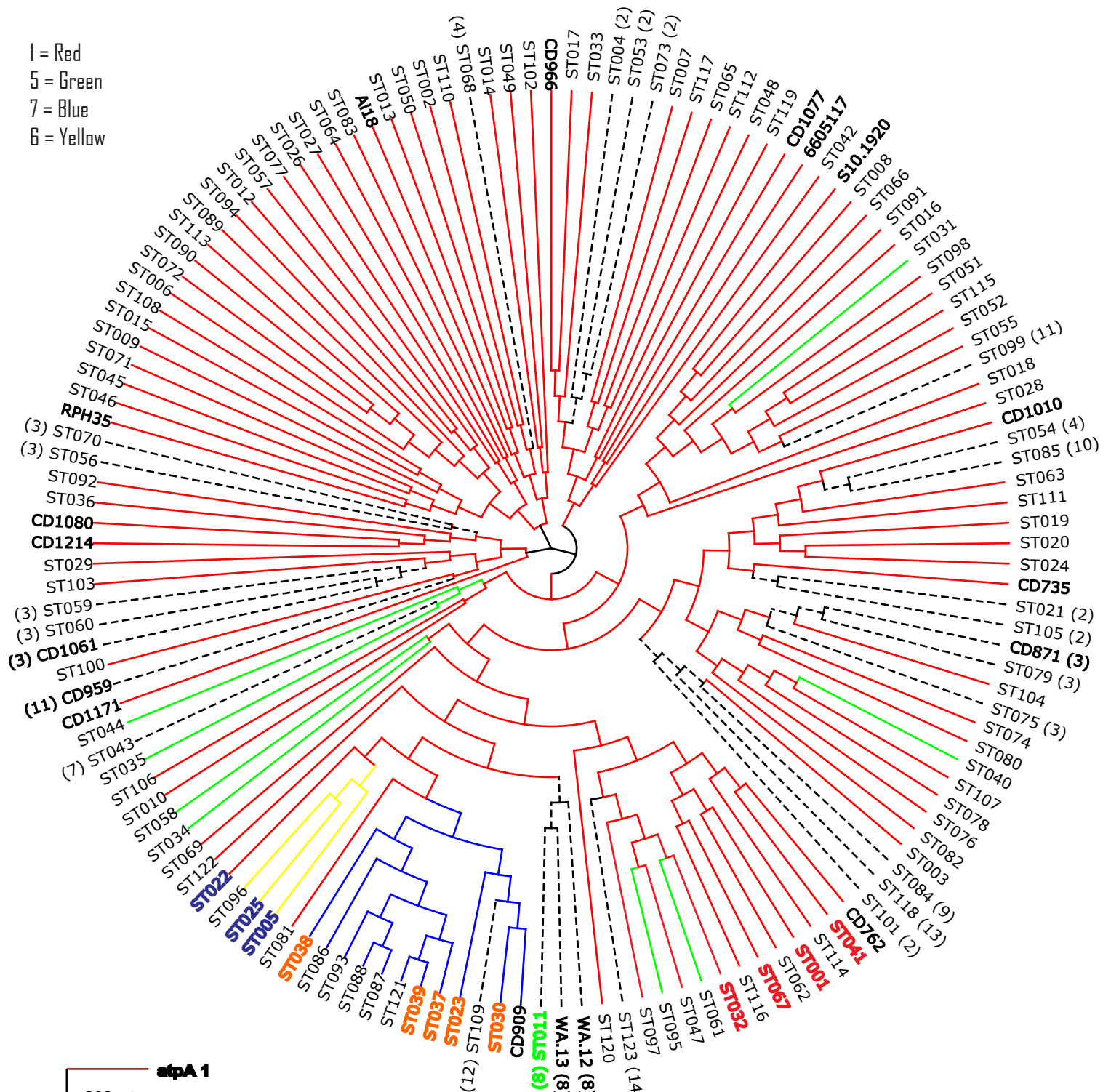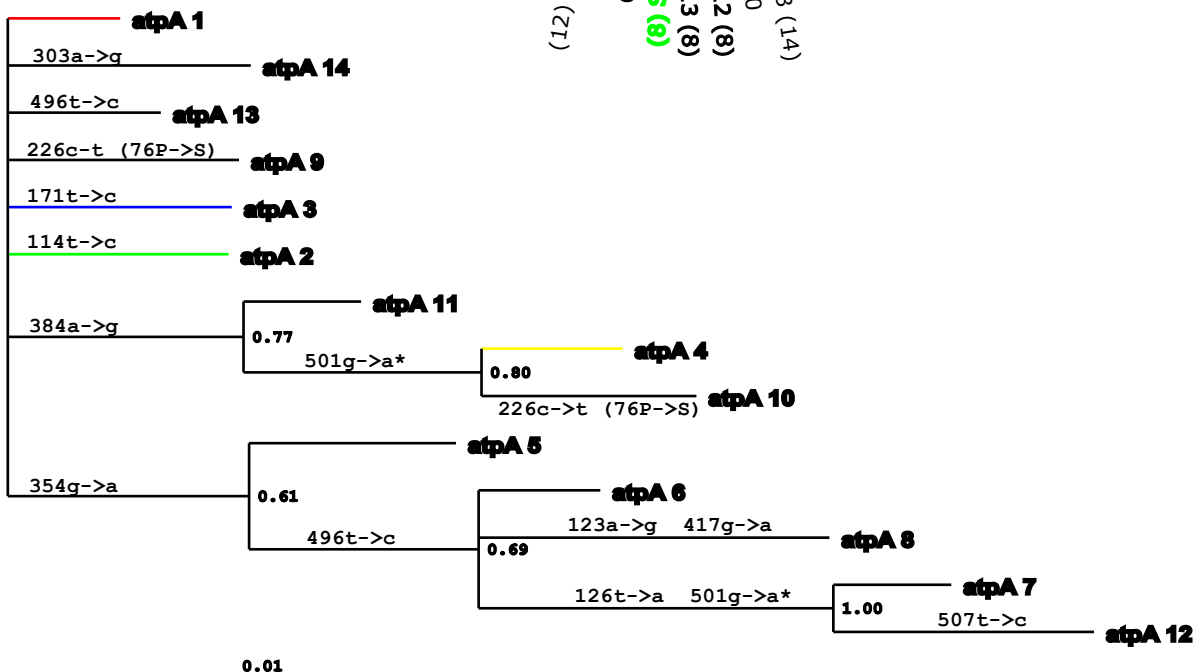

Supplement: Figure S2 — MAFFT circular phylogram with atp alleles. A) Circle phylogeny coloured by allele. B) MrBayes SNP phylogram of atp alleles coloured by allele. SNPs indicated on branch. * indicates a non-unique SNP that occurs in more than one place on phylogram. (PDF) [file pone.0031559.s002.pdf]

2 = Red  
 6 = Green  
 7 = Blue  
 3 = Yellow

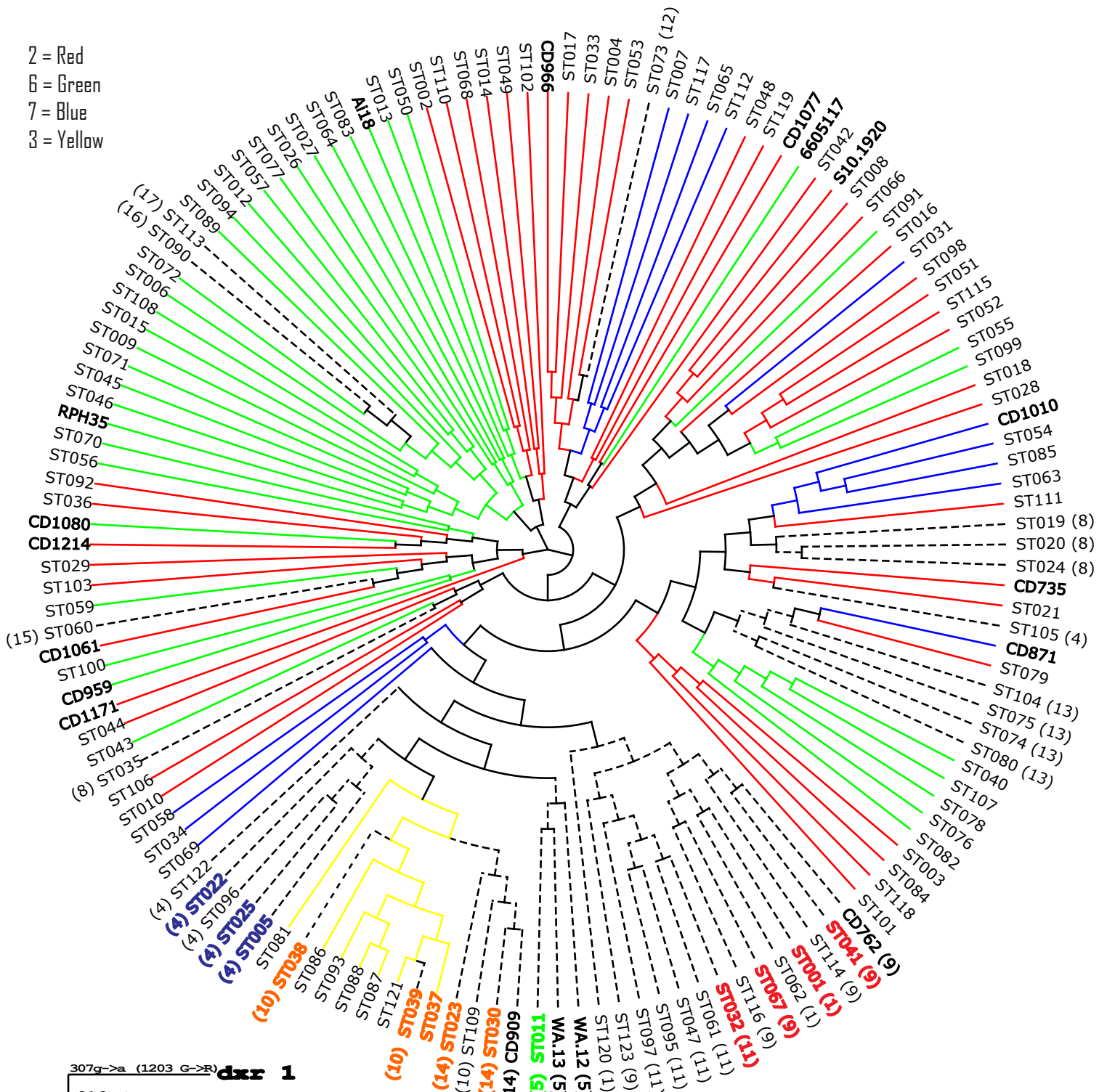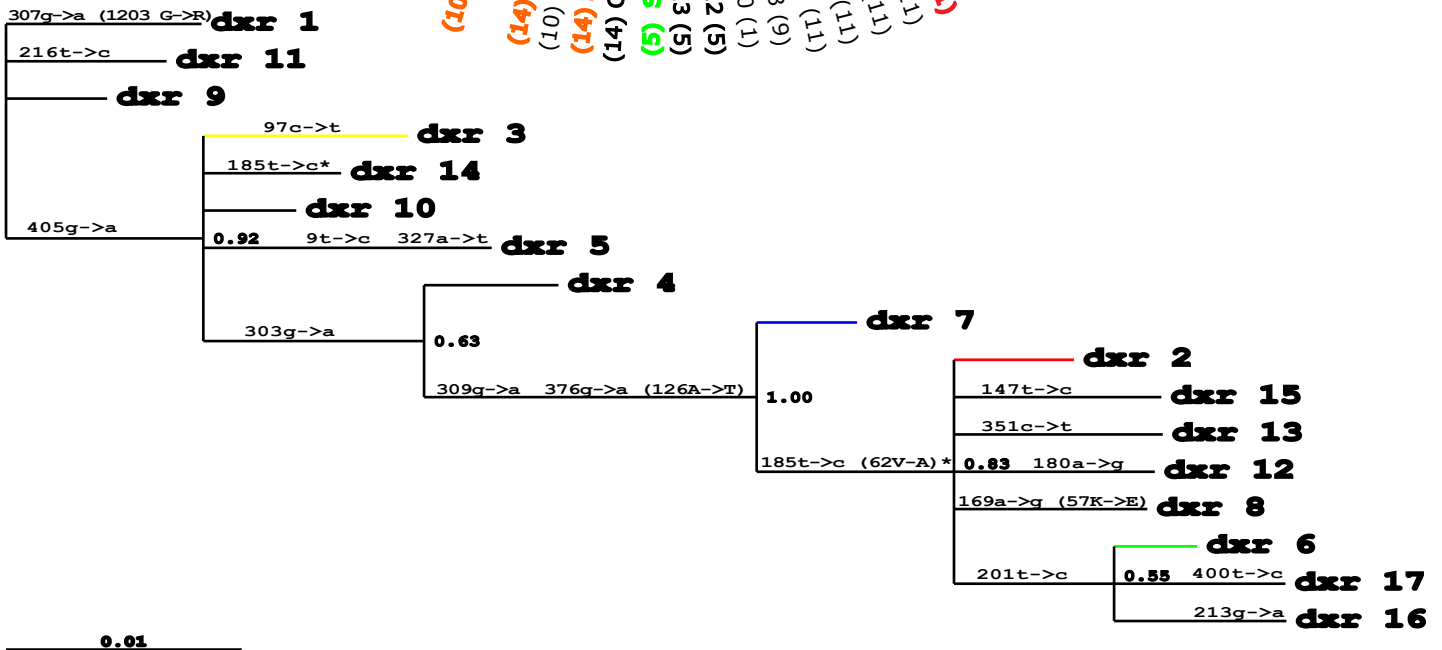

Supplement: Figure S3 — MAFFT circular phylogram with dxr alleles. A) Circle phylogeny coloured by allele. B) MrBayes SNP phylogram of dxr alleles coloured by allele. SNPs indicated on branch. * indicates a non-unique SNP that occurs in more than one place on phylogram. (PDF) [file pone.0031559.s003.pdf]

1 = Red  
5 = Green  
6 = Blue  
8 = Yellow

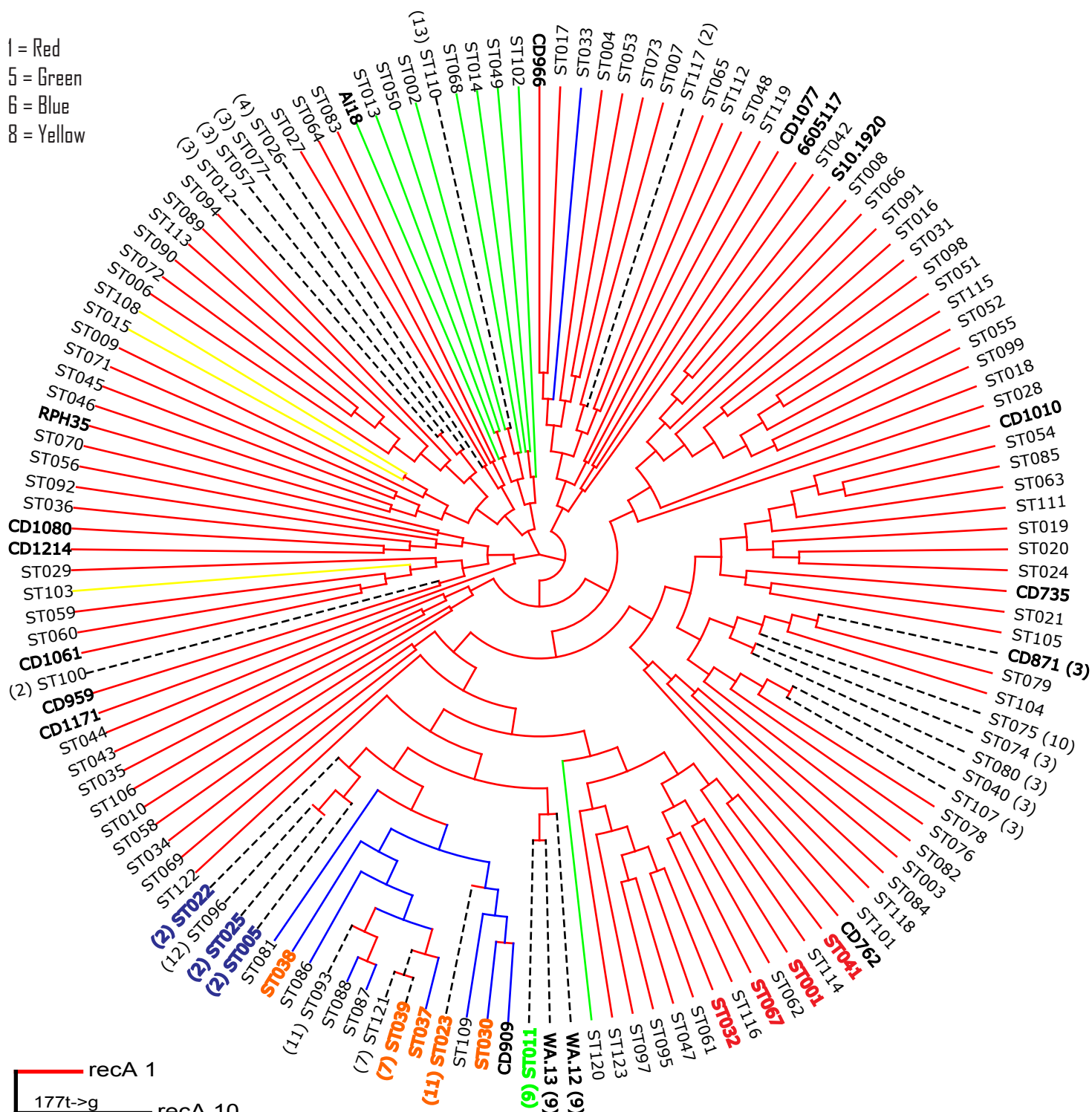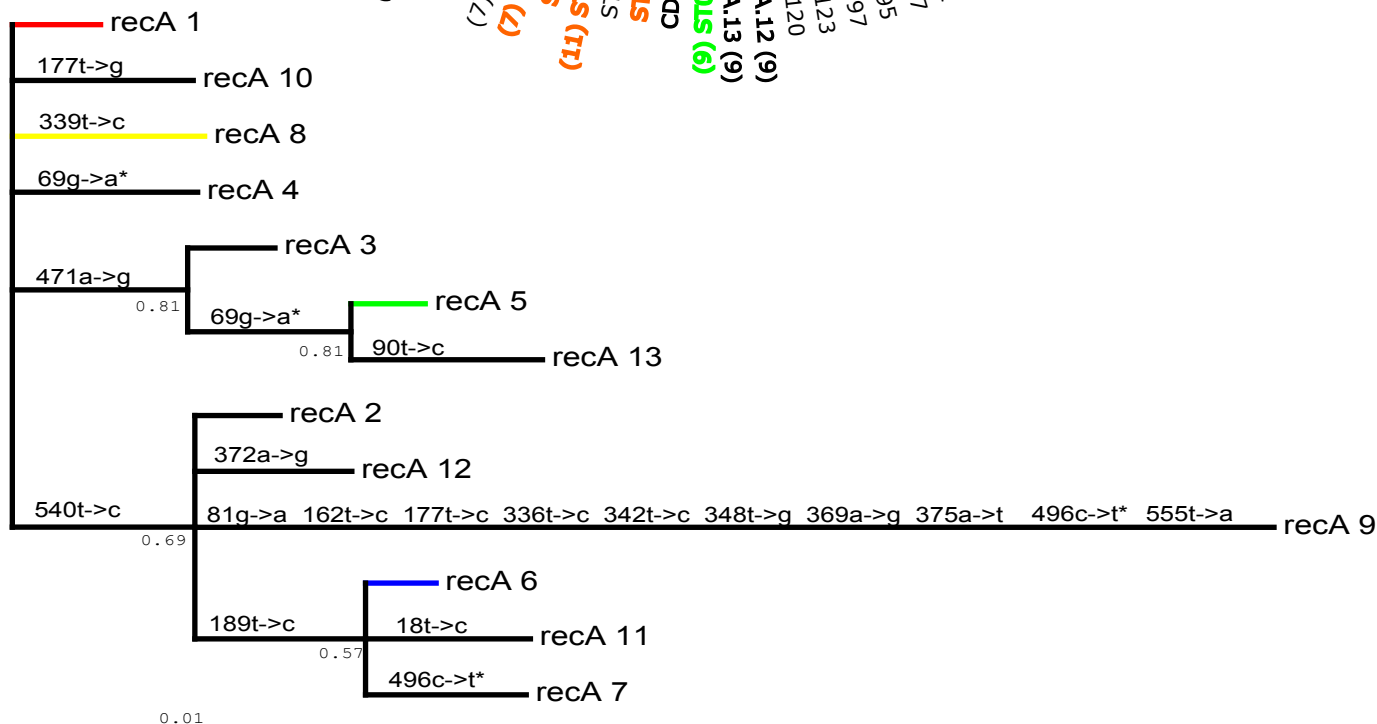

Supplement: Fgure S5 — MAFFT circular phylogram with recA alleles. A) Circle phylogeny coloured by allele. B) MrBayes SNP phylogram of recA alleles coloured by allele. SNPs indicated on branch. * indicates a non-unique SNP that occurs in more than one place on phylogram. (PDF) [file pone.0031559.s005.pdf]

3 = Red  
5 = Green  
1 = Blue  
7 = Yellow

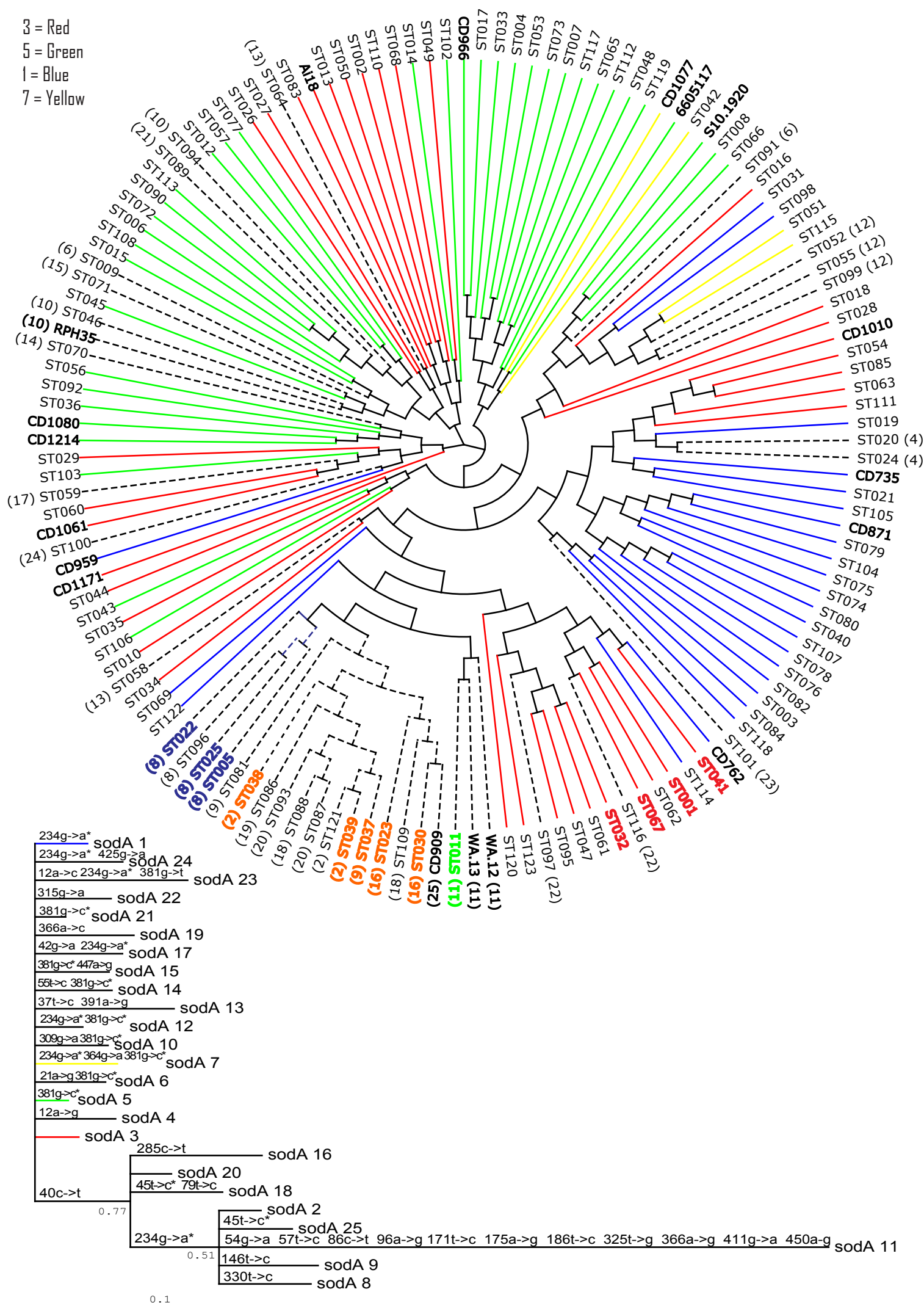

Supplement: Figure S6 — MAFFT circular phylogram with sodA alleles. A) Circle phylogeny coloured by allele. B) MrBayes SNP phylogram of sodA alleles coloured by allele. SNPs indicated on branch. * indicates a non-unique SNP that occurs in more than one place on phylogram. (PDF) [file pone.0031559.s006.pdf]

1 = Red  
 3 = Green  
 6 = Blue  
 10 = Yellow

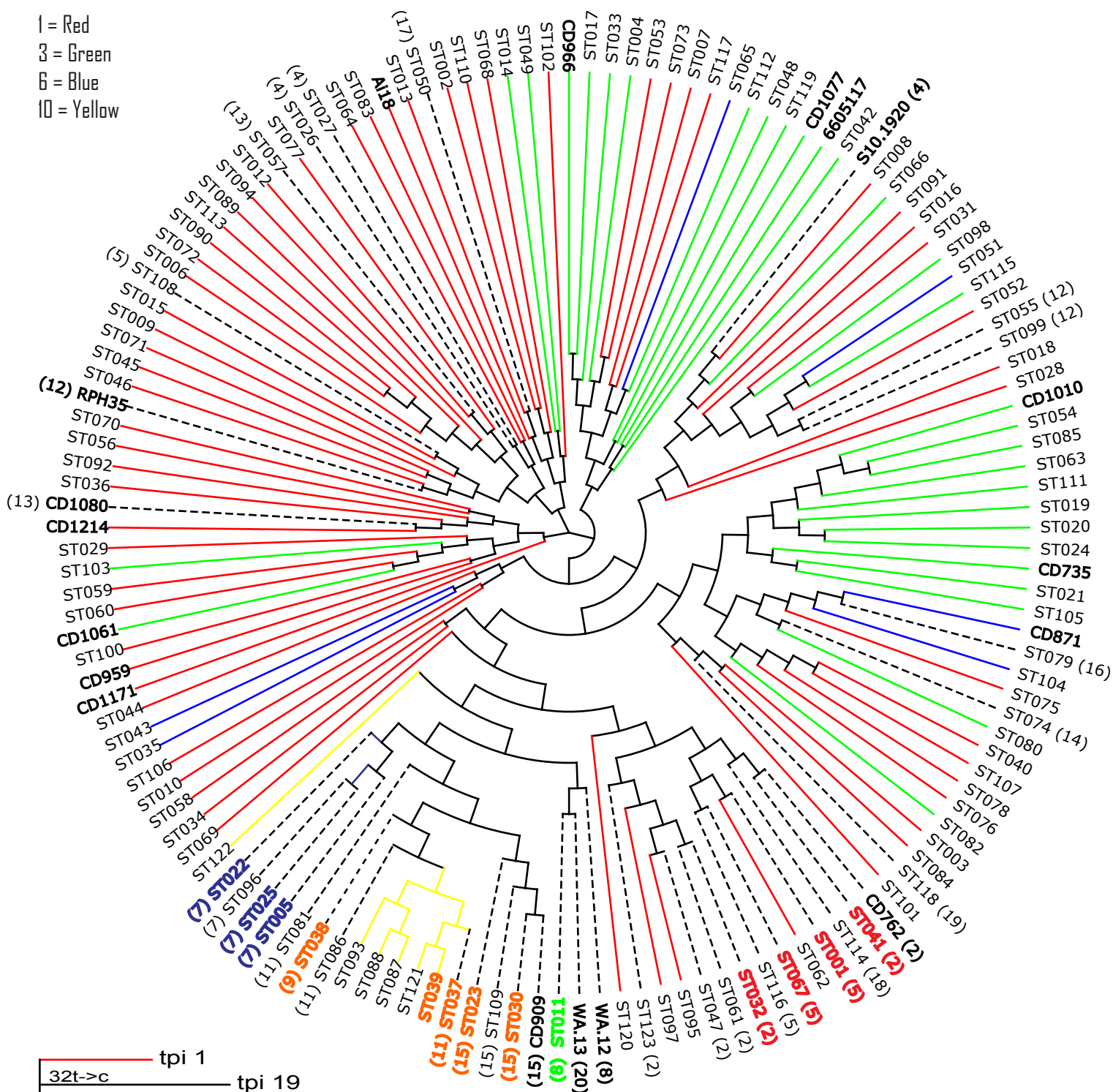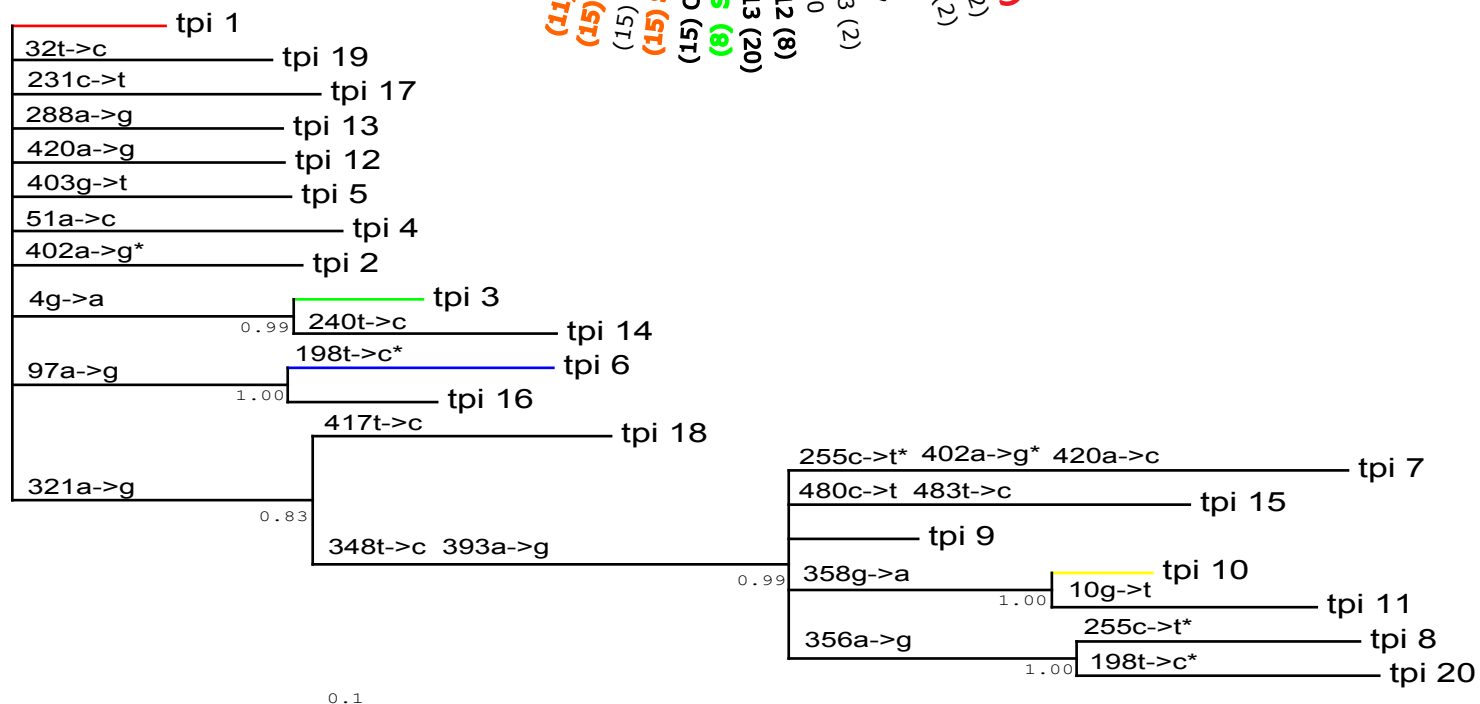

Supplement: Figure S7 — MAFFT circular phylogram with tpi alleles. A) Circle phylogeny coloured by allele. B) MrBayes SNP phylogram of tpi alleles coloured by allele. SNPs indicated on branch. * indicates a non-unique SNP that occurs in more than one place on phylogram. (PDF) [file pone.0031559.s007.pdf]

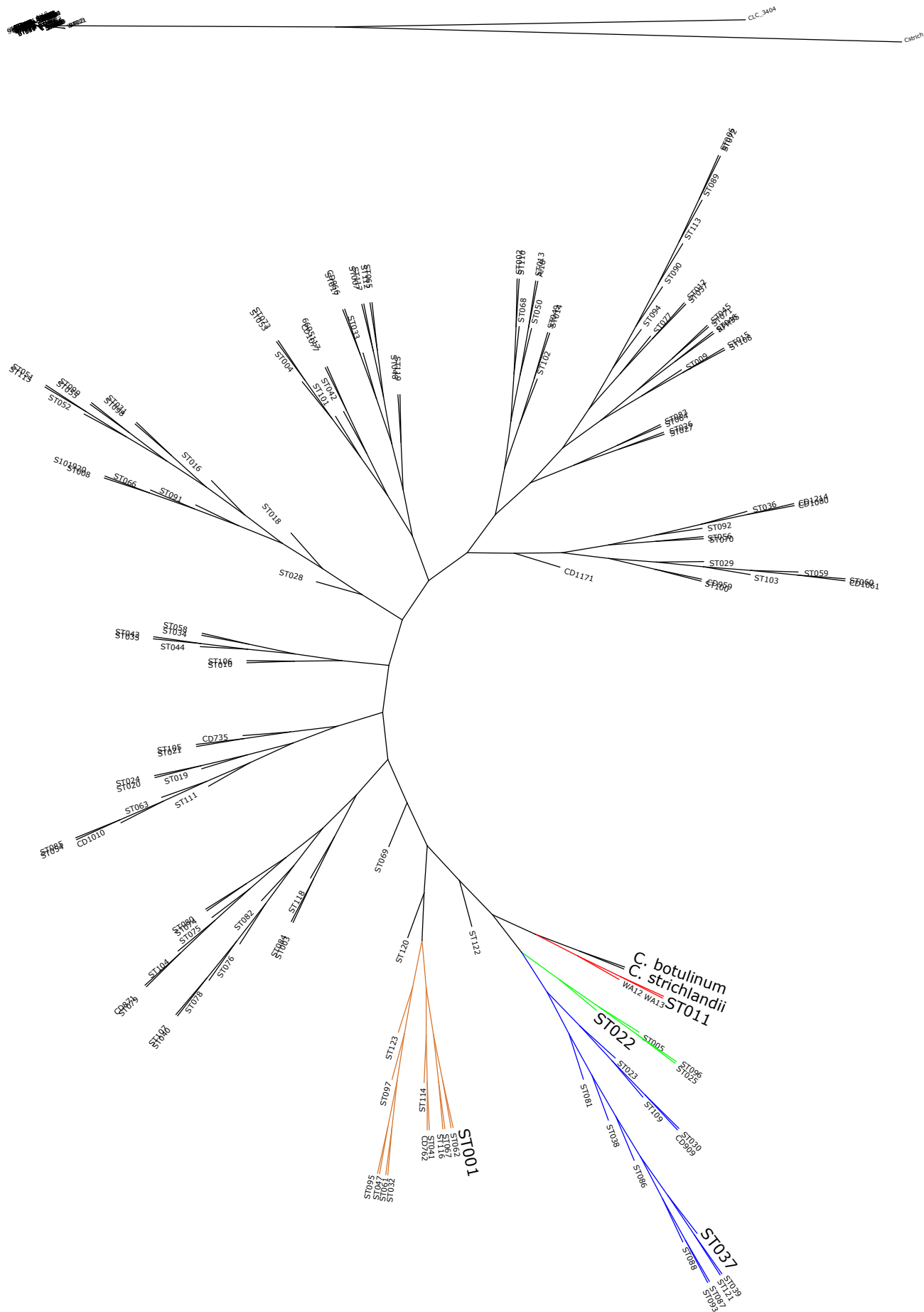

Supplement: Figure S8 — MAFFT Rooted phylogram. A) Scaled phylogram displaying relationship of C. botulinum A BoNT/A1 (ATCC 19397) and C. strichlandii DSM 519 to C. difficile, B) MAFFT unscaled phylogram showing location of C. botulinum A BoNT/A1 (ATCC 19397) and C. strichlandii DSM 519 within C. difficile population. Black branches = clade 1, orange branches = clade 2 (inc ST-1), green ranches = clade 3 (inc ST-23) blue branches = clade 4 (inc ST-37), red branches = clade 5 (inc ST-11). (PDF) [file pone.0031559.s008.pdf]
