# Supplementary material for: Macro and Micro Diversity of Clostridium difficile Isolates from Diverse Sources and Geographical Locations
Source: PLoS One. 2012 Mar 2;7(3):e31559. doi: 10.1371/journal.pone.0031559 (PMC3292544; doi:10.1371/journal.pone.0031559)
Supplement: Figure S4 — MAFFT circular phylogram with glyA alleles. A) Circle phylogeny coloured by allele. B) MrBayes SNP phylogram of glyA alleles coloured by allele. SNPs indicated on branch. * indicates a non-unique SNP that occurs in more than one place on phylogram. ! indicates a SNPs in glyA04 which has reverted to ancestral in glyA03 and into a non-synonymous SNP in glyA14. (PDF) [file pone.0031559.s004.pdf]

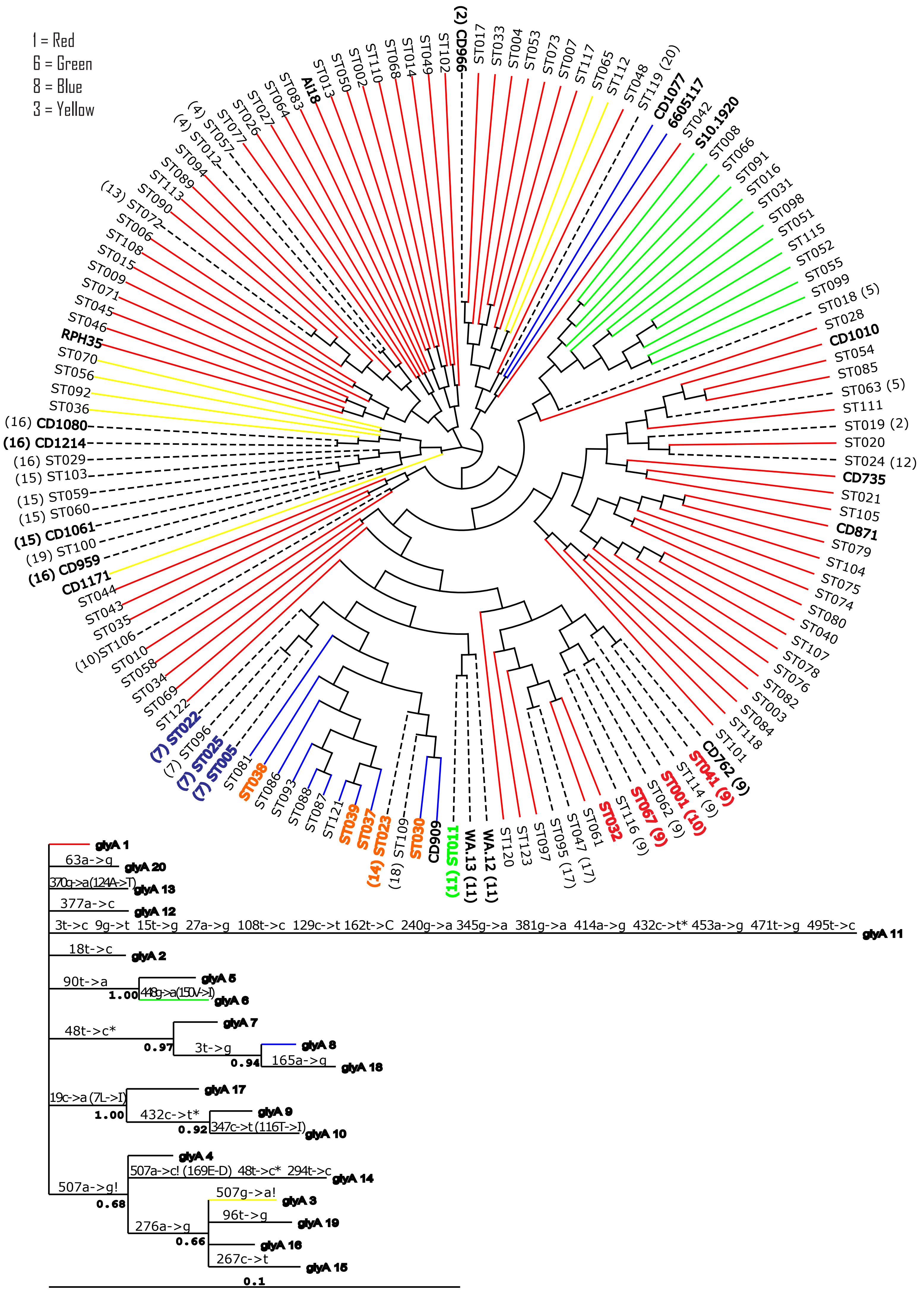

1 = Red
6 = Green
8 = Blue
3 = Yellow
(2) CD966
ST017
ST033
ST004
ST053
ST073
ST007
ST117
ST065
ST112
ST048
ST119 (20)
CD1077
6605117
ST042
S10.1920
ST008
ST066
ST091
ST016
ST031
ST098
ST051
ST115
ST052
ST055
ST099
ST018 (5)
ST028
CD1010
ST054
ST085
ST063 (5)
ST111
ST019 (2)
ST020
ST024 (12)
CD735
ST021
ST105
CD871
ST079
ST104
ST075
ST074
ST080
ST040
ST107
ST078
ST076
ST082
ST003
ST084
ST118
ST101
CD762 (9)
ST041 (9)
ST114 (9)
ST001 (10)
ST062 (9)
ST067 (9)
ST116 (9)
ST032
ST061
ST047 (17)
ST095 (17)
ST097
ST123
ST120
WA.12 (11)
WA.13 (11)
(11) ST011
CD909
ST030
(18) ST109
(14) ST023
ST037
ST039
ST121
ST087
ST088
ST093
ST086
ST038
ST081
(7) ST005
(7) ST025
(7) ST096
(7) ST022
ST122
ST069
ST034
ST058
ST010
(10)ST106
ST035
ST043
ST044
CD1171
(16) CD959
(19) ST100
(15) CD1061
(15) ST060
(15) ST059
(15) ST103
(16) ST029
(16) CD1214
(16) CD1080
ST036
ST092
ST056
ST070
RPH35
ST046
ST045
ST071
ST009
ST015
ST108
ST006
(13) ST072
ST090
ST113
ST089
ST094
(4) ST012
(4) ST057
ST077
ST026
ST027
ST064
ST083
AI18
ST013
ST050
ST002
ST110
ST068
ST014
ST049
ST102
glyA 1
63a->g
glyA 20
370g->a(124A->T)
glyA 13
377a->c
glyA 12
3t->c 9g->t 15t->g 27a->g 108t->c 129c->t 162t->C 240g->a 345g->a 381g->a 414a->g 432c->t* 453a->g 471t->g 495t->c
glyA 11
18t->c
glyA 2
90t->a
glyA 5
1.00
448g->a(150V->I)
glyA 6
48t->c*
glyA 7
0.97
3t->g
glyA 8
0.94
165a->g
glyA 18
19c->a (7L->I)
glyA 17
1.00
432c->t*
glyA 9
0.92
347c->t (116T->I)
glyA 10
glyA 4
507a->c! (169E-D) 48t->c* 294t->c
glyA 14
507a->g!
0.68
507g->a!
glyA 3
96t->g
glyA 19
276a->g
0.66
glyA 16
267c->t
glyA 15
0.1
