# Supplementary material for: Macro and Micro Diversity of Clostridium difficile Isolates from Diverse Sources and Geographical Locations
Source: PLoS One. 2012 Mar 2;7(3):e31559. doi: 10.1371/journal.pone.0031559 (PMC3292544; doi:10.1371/journal.pone.0031559)
Supplement: Data S1 — Strains in study (inc MLST data). (PDF) [file pone.0031559.s009.pdf]

Strain	Ribotype	ST group	adk	atpA	dxr	glyA	recA	sodA	tpi	ST	Origin	Location	Date isolated
630	RT012	grp 1	**1**	4	7	1	1	3	3	ST54	Human	Switzerland	1982
IS58	RT033	grp 5	5	8	5	11	9	11	8	ST11	Human	Australia	before 2004
2046	RT001	grp 1	**1**	1	2	1	1	1	1	ST03			
2047	NT	grp 1	**1**	1	2	6	1	5	1	ST08			
2050	RT015	grp 1	2	5	2	1	1	3	1	ST44			
2052	RT023	grp 3	**1**	**6**	4	7	2	8	7	ST05			
2053	RT027	grp 2	**1**	1	1	10	1	3	5	ST01			
2054	NT	grp 5	5	**8**	5	11	9	11	8	ST11			
2055	RT106	grp 1	**1**	1	2	1	1	7	1	ST42			
5342	NT	grp 5	5	8	5	11	9	11	8	ST11	Bovine	USA	
5350	RT078	grp 5	5	8	5	11	9	11	8	ST11	Bovine	USA	
5359	NT	grp 2	1	1	1	10	1	3	5	ST01			
5361	RT078	grp 5	5	8	5	11	9	11	8	ST11	Bovine	USA	26/12/2006
5363	RT078	grp 5	5	8	5	11	9	11	8	ST11	Bovine	USA	20/03/2007
5370	RT027	grp 2	1	1	1	10	1	3	5	ST01	Bovine	USA	26/06/2007
5373	RT027	grp 2	1	1	1	10	1	3	5	ST01	Bovine	USA	20/03/2007
5379	NT	grp 5	5	8	5	11	9	11	8	ST11	Bovine	USA	
5384	RT078	grp 5	5	8	5	11	9	11	8	ST11	Bovine	USA	20/03/2007
5397	RT078	grp 5	5	8	5	11	9	11	8	ST11	Bovine	USA	26/06/2007
5407	RT078	grp 5	5	8	5	11	9	11	8	ST11	Bovine	USA	26/09/2006
5408	RT078	grp 5	5	8	5	11	9	11	8	ST11	Bovine	USA	26/06/2007
5416	NT	grp 5	5	8	5	11	9	11	8	ST11	Bovine	USA	2006
5416	RT078	grp 5	5	8	5	11	9	11	8	ST11	Bovine	USA	2006
5427	RT027	grp 2	1	1	1	10	1	3	5	ST01	Bovine	USA	31/07/2007
5428	RT078	grp 5	5	8	5	11	9	11	8	ST11	Bovine	USA	20/03/2007
5429	RT078	grp 5	5	8	5	11	9	11	8	ST11	Bovine	USA	15/10/2007
5432	RT078	grp 5	5	8	5	11	9	11	8	ST11	Bovine	USA	23/01/2007
5444	RT078	grp 5	5	8	5	11	9	11	8	ST11	Bovine	USA	15/10/2007
5468	NT	grp 5	5	8	5	11	9	11	8	ST11	Bovine	USA	
5898	RT078	grp 5	5	8	5	11	9	11	8	ST11	Bovine	USA	20/03/2007
5904	RT078	grp 5	5	8	5	11	9	11	8	ST11	Bovine	USA	15/10/2007
5911	RT078	grp 5	5	8	5	11	9	11	8	ST11	Bovine	USA	17/04/2007
5912	NT	grp 5	5	8	5	11	9	11	8	ST11	Bovine	USA	
5917	RT078	grp 5	5	8	5	11	9	11	8	ST11	Bovine	USA	15/10/2007
5920	NT	grp 1	1	1	2	1	1	1	1	ST03	Bovine	USA	31/07/2007
5921	RT078	grp 5	5	8	5	11	9	11	8	ST11	Bovine	USA	20/11/2006
5927	NT	grp 5	5	8	5	11	9	11	8	ST11	Bovine	USA	
5927	NT	grp 5	5	8	5	11	9	11	8	ST11	Bovine	USA	
5933	NT	grp 5	5	8	5	11	9	11	8	ST11	Bovine	USA	
5938	RT078	grp 5	5	8	5	11	9	11	8	ST11	Bovine	USA	20/11/2006
5946	RT078	grp 5	5	8	5	11	9	11	8	ST11	Bovine	USA	20/03/2007

5954	NT	grp 5	5	8	5	11	9	11	8	ST11	Bovine	USA	
5963	RT078	grp 5	5	8	5	11	9	11	8	ST11	Bovine	USA	20/11/2006
5968	RT078	grp 5	5	8	5	11	9	11	8	ST11	Bovine	USA	20/03/2007
5982	RT078	grp 5	5	8	5	11	9	11	8	ST11	Bovine	USA	20/11/2006
5983	RT078	grp 5	5	8	5	11	9	11	8	ST11	Bovine	USA	20/03/2007
5984	RT078	grp 5	5	8	5	11	9	11	8	ST11	Bovine	USA	15/10/2007
5986	RT078	grp 5	5	8	5	11	9	11	8	ST11	Bovine	USA	31/07/2007
5987	RT078	grp 5	5	8	5	11	9	11	8	ST11	Bovine	USA	15/10/2007
5992	NT	grp 5	5	8	5	11	9	11	8	ST11	Bovine	USA	
5996	RT078	grp 5	5	8	5	11	9	11	8	ST11	Bovine	USA	15/10/2007
6004	NT	grp 5	5	8	5	11	9	11	8	ST11	Bovine	USA	
6005	RT078	grp 5	5	8	5	11	9	11	8	ST11	Bovine	USA	26/06/2007
6007	RT078	grp 5	5	8	5	11	9	11	8	ST11	Bovine	USA	2007
6007	RT078	grp 5	5	8	5	11	9	11	8	ST11	Bovine	USA	2007
6014	RT027	grp 2	1	1	1	10	1	3	5	ST01	Bovine	USA	
6015	RT078	grp 5	5	8	5	11	9	11	8	ST11	Bovine	USA	15/10/2007
6021	RT078	grp 5	5	8	5	11	9	11	8	ST11	Bovine	USA	20/03/2007
6065	NT	grp 5	5	8	5	11	9	11	8	ST11	Bovine	USA	
6067	NT	grp 5	5	8	5	11	9	11	8	ST11	Bovine	USA	
8864	RT036	grp 2	1	1	1	9	1	3	1	ST62	Human	UK	before 2004
10/33	RT078	grp 5	5	8	5	11	9	11	8	ST11			
30256	NT	grp 5	5	8	5	11	9	11	8	ST11	Bovine	USA	
31807	NT	grp 5	5	8	5	11	9	11	8	ST11			
13/03	RT078	grp 5	5	8	5	11	9	11	8	ST11			
19/07	RT078	grp 5	5	8	5	11	9	11	8	ST11			
19/09	RT078	grp 5	5	8	5	11	9	11	8	ST11			
17/10	RT078	grp 5	5	8	5	11	9	11	8	ST11			
3/11	RT078	grp 5	5	8	5	11	9	11	8	ST11			
80249	RT078	grp 5	5	8	5	11	9	11	8	ST11			20/03/2007
2004101	RT027	grp 2	1	1	1	10	1	3	5	ST01	Human	USA	2004
2004102	RT027	grp 2	1	1	1	10	1	3	5	ST01	Human	USA	2004
2004118	RT027	grp 2	1	1	1	10	1	3	5	ST01	Human	USA	2004
2004163	RT027	grp 2	1	1	1	10	1	3	5	ST01	Human	USA	2004
2005079	NT	grp 2	1	1	1	10	1	3	5	ST01	Human	USA	2005
2005088	NT	grp 5	5	8	5	11	9	11	8	ST11	Porcine	USA	2005
2005093	RT126	grp 5	5	8	5	11	9	11	8	ST11	Porcine	USA	2005
2005094	NT	grp 5	5	8	5	11	9	11	8	ST11	Porcine	USA	2005
2005325	RT078	grp 5	5	8	5	11	9	11	8	ST11	Human	USA	2005
2005508	NT	grp 5	5	8	5	11	9	11	8	ST11	Porcine	USA	2005
2005511	RT078	grp 5	5	8	5	11	9	11	8	ST11	Porcine	USA	2005
2005515	RT078	grp 5	5	8	5	11	9	11	8	ST11	Porcine	USA	2005
2005517	NT	grp 1	1	1	2	1	1	5	1	ST48	Porcine	USA	2005

2005519	NT	grp 5	5	8	5	11	9	11	8	ST11	Porcine	USA	2005
2006237	RT027	grp 2	1	1	1	10	1	3	5	ST01	Equine	USA	2006
2006238	RT078	grp 5	5	8	5	11	9	11	8	ST11	Bovine	USA	2006
2006239	RT078	grp 5	5	8	5	11	9	11	8	ST11	Bovine	USA	2006
2006240	RT078	grp 5	5	8	5	11	9	11	8	ST11	Bovine	USA	2006
2006241	RT078	grp 5	5	8	5	11	9	11	8	ST11	Bovine	USA	2006
2006243	RT078	grp 5	5	8	5	11	9	11	8	ST11	Bovine	USA	2006
2006244	RT078	grp 5	5	8	5	11	9	11	8	ST11	Bovine	USA	2006
2006245	RT078	grp 5	5	8	5	11	9	11	8	ST11	Bovine	USA	2006
2006246	RT078	grp 5	5	8	5	11	9	11	8	ST11	Bovine	USA	2006
2006253	RT078	grp 5	5	8	5	11	9	11	8	ST11	Bovine	USA	2006
2006254	RT078	grp 5	5	8	5	11	9	11	8	ST11	Bovine	USA	2006
2006354	RT078	grp 5	5	8	5	11	9	11	8	ST11	Human	USA	2006
2006379	RT078	grp 5	5	8	5	11	9	11	8	ST11	Human	USA	2006
2006437	RT078	grp 5	5	8	5	11	9	11	8	ST11	Food	USA	2006
2006438	RT078	grp 5	5	8	5	11	9	11	8	ST11	Food	USA	2006
2006439	RT027	grp 2	1	1	1	10	1	3	5	ST01	Food	USA	2006
2006460	RT078	grp 5	5	8	5	11	9	11	8	ST11	Human	USA	2006
2007007	RT078	grp 5	5	8	5	11	9	11	8	ST11	Human	USA	2007
2007011	RT078	grp 5	5	8	5	11	9	11	8	ST11	Human	USA	2007
2007019	RT126	grp 5	5	8	5	11	9	11	8	ST11	Human	USA	2007
2007024	RT078	grp 5	5	8	5	11	9	11	8	ST11	Human	USA	2007
2007042	RT027	grp 2	1	1	1	10	1	3	5	ST01	Food	USA	2007
2007054	RT078	grp 5	5	8	5	11	9	11	8	ST11	Human	USA	2007
2007098	NT	grp 2	1	1	11	1	1	3	2	ST32	Human	USA	2007
2007134	NT	grp 5	5	8	5	11	9	11	8	ST11	Human	USA	2007
2007140	RT027	grp 2	1	1	1	10	1	3	5	ST01	Human	USA	2007
2007195	NT	grp 2	1	1	9	9	1	3	2	ST41	Human	USA	2007
2007206	NT	grp 5	5	8	5	11	9	11	8	ST11	Human	USA	2007
2007218	RT027	grp 2	1	1	1	10	1	3	5	ST01	Food	USA	2007
2007219	RT078	grp 5	5	8	5	11	9	11	8	ST11	Food	USA	2007
2007222	RT027	grp 2	1	1	1	10	1	3	5	ST01	Food	USA	2007
2007223	RT027	grp 2	1	1	1	10	1	3	5	ST01	Food	USA	2007
2007224	NT	grp 5	5	8	5	11	9	11	8	ST11	Food	USA	2007
2007229	RT126	grp 5	5	8	5	11	9	11	8	ST11	Food	USA	2007
2007230	RT078	grp 5	5	8	5	11	9	11	8	ST11	Food	USA	2007
2007235	NT	grp 2	1	1	1	10	1	3	5	ST01	Food	USA	2007
2007334	RT078	grp 5	5	8	5	11	9	11	8	ST11	Human	USA	2007
2007361	RT078	grp 5	5	8	5	11	9	11	8	ST11	Human	USA	2007
2007380	RT078	grp 5	5	8	5	11	9	11	8	ST11	Human	USA	2007
2007600	RT126	grp 5	5	8	5	11	9	11	8	ST11	Human	Spain	2007
2007601	RT126	grp 5	5	8	5	11	9	11	8	ST11	Human	Spain	2007

2007606	RT078	grp 5	5	8	5	11	9	11	8	ST11	Food	USA	2007
2007607	RT078	grp 5	5	8	5	11	9	11	8	ST11	Food	USA	2007
2007786	RT078	grp 5	5	8	5	11	9	11	8	ST11	Human	Italy	2007
2007792	RT078	grp 5	5	8	5	11	9	11	8	ST11	Human	Italy	2007
2007825	RT027	grp 2	1	1	1	10	1	3	5	ST01	Human	USA	2007
2007826	NT	grp 2	1	5	11	1	1	3	2	ST61	Human	USA	2007
2007827	RT262	grp 2	1	1	9	9	1	3	5	ST67	Human	USA	2007
2007828	NT	grp 2	1	1	1	10	1	3	5	ST01	Human	USA	2007
2007829	RT262	grp 2	1	1	9	9	1	3	5	ST67	Human	USA	2007
2007830	RT027	grp 2	1	1	1	10	1	3	5	ST01	Human	USA	2007
2007831	RT001	grp 1	1	1	2	1	1	1	1	ST03	Human	USA	2007
2007832	RT027	grp 2	1	1	1	10	1	3	5	ST01	Human	USA	2007
2007833	RT027	grp 2	1	1	1	10	1	3	5	ST01	Human	USA	2007
2007834	RT078	grp 5	5	8	5	11	9	11	8	ST11	Human	USA	2007
2007835	RT078	grp 5	5	8	5	11	9	11	8	ST11	Human	USA	2007
2007838	RT078	grp 5	5	8	5	11	9	11	8	ST11	Human	USA	2007
2007839	NT	grp 2	1	1	1	10	1	3	5	ST01	Human	USA	2007
2007841	NT	grp 1	1	1	2	1	1	1	1	ST03	Human	USA	2007
2007843	NT	grp 2	1	5	11	1	1	3	2	ST61	Food	USA	2007
2007844	NT	grp 2	1	5	11	1	1	3	2	ST61	Food	USA	2007
2007850	RT027	grp 2	1	1	1	10	1	3	5	ST01	Household	USA	2007
2007855	RT027	grp 2	1	1	1	10	1	3	5	ST01	Bovine	USA	2007
6600639	RT078	grp 5	**5**	8	5	11	9	11	8	ST11	Human	UK	
6600726	RT050	grp 1	**1**	**1**	2	5	1	3	1	ST18	Human	UK	
6601158	NT	grp 1	1	1	2	1	1	5	3	ST17	Human	UK	
6601667	RT002	grp 1	**1**	1	2	6	1	5	1	ST08	Human	UK	
6603036	RT015	grp 1	**2**	1	2	1	1	3	1	ST10	Human	UK	
6603061	NT	grp 1	1	1	2	1	5	3	1	ST02	Human	UK	
6604395	RT015	grp 1	**2**	5	2	1	1	3	1	ST44	Human	UK	
6605117	RT005	grp 1	2	1	6	8	1	5	1	New (ST131)	Human	UK	
6605475	RT015	grp 1	2	5	2	1	1	3	1	ST44	Human	UK	
6614282	RT023	grp 3	1	6	4	7	2	8	7	ST05	Human	UK	
6614376	RT078	grp 5	**5**	**8**	5	11	9	11	8	ST11	Human	UK	
6615591	RT002	grp 1	**1**	1	2	6	1	5	1	ST08	Human	UK	
6615723	RT014	grp 1	**1**	1	2	1	5	3	3	ST49	Human	UK	
6616023	RT078	grp 5	**5**	**8**	5	11	9	11	8	ST11	Human	UK	
6616104	RT078	grp 5	**5**	8	5	11	9	11	8	ST11	Human	UK	
001-01	RT001	grp 1	**1**	1	2	1	1	1	1	ST03		UK	
001-02	RT001	grp 1	**1**	**1**	2	1	1	1	1	ST03		UK	
001-03	RT001	grp 1	**1**	**1**	2	1	1	1	1	ST03		UK	
001-04	RT001	grp 1	**1**	1	2	1	1	1	1	ST03		UK	
001-05	RT001	grp 1	**1**	1	2	1	1	1	1	ST03		UK	

001-06	RT001	grp 1	**1**	1	2	1	1	1	1	ST03		UK	
001-07	RT001	grp 1	**1**	**1**	2	1	1	1	1	ST03		UK	
001-08	RT001	grp 1	**1**	1	2	1	5	3	1	ST02		UK	
001-09	RT001	grp 1	**1**	1	2	1	1	1	1	ST03		UK	
001-10	RT001	grp 1	**1**	1	2	1	1	1	1	ST03		UK	
078W	RT078	grp 5	5	8	5	11	9	11	8	ST11			
106- 01	RT106	grp 1	**1**	1	2	1	1	7	1	ST42	Human	UK	
106- 02	RT106	grp 1	**1**	**1**	2	1	1	7	1	ST42	Human	UK	
106- 04	RT106	grp 1	**1**	**1**	2	1	1	7	1	ST42	Human	UK	
106- 05	RT106	grp 1	**1**	**1**	2	1	1	7	1	ST42	Human	UK	
106- 06	RT106	grp 1	1	1	2	1	1	7	1	ST42	Human	UK	
106-07	RT106	grp 1	1	1	2	1	1	7	1	ST42	Human	UK	
106-10	RT106	grp 1	1	1	2	1	1	7	1	ST42	Human	UK	
17/50	RT078	grp 5	5	8	5	11	9	11	8	ST11			
18/21	RT078	grp 5	5	8	5	11	9	11	8	ST11			
19/44	RT078	grp 5	5	8	5	11	9	11	8	ST11			
19/52	RT078	grp 5	5	8	5	11	9	11	8	ST11			
19/72	RT078	grp 5	5	8	5	11	9	11	8	ST11			
20/28	RT078	grp 5	5	8	5	11	9	11	8	ST11			
22/31	RT078	grp 5	5	8	5	11	9	11	8	ST11			
23/41	RT078	grp 5	5	8	5	11	9	11	8	ST11			
25/40	RT078	grp 5	5	8	5	11	9	11	8	ST11			
3623 -03	NT	grp 1	1	1	6	1	4	3	4	ST26	Human	Germany	before 2004
5353 (3/20)	RT078	grp 5	5	8	5	11	9	11	8	ST11	Bovine	USA	20/03/2007
5354 (17/4)	RT027	grp 2	1	1	1	10	1	3	5	ST01	Bovine	USA	
5379 (26/12)	RT078	grp 5	5	8	5	11	9	11	8	ST11	Bovine	USA	
5404 (17/4)	RT078	grp 5	5	8	5	11	9	11	8	ST11	Bovine	USA	17/04/2007
5404 (9/26)	RT078	grp 5	5	8	5	11	9	11	8	ST11	Bovine	USA	26/09/2006
5424 (2/20)	RT078	grp 5	5	8	5	11	9	11	8	ST11	Bovine	USA	20/02/2007
5945 (2/21)	NT	grp 5	5	8	5	11	9	11	8	ST11	Bovine	USA	20/11/2006
5964 (20/11)	RT078	grp 5	5	8	5	11	9	11	8	ST11	Bovine	USA	20/11/2006
5964 (9/1)	RT078	grp 5	5	8	5	11	9	11	8	ST11	Bovine	USA	09/01/2007
5994 (9/4)	NT	grp 5	5	8	5	11	9	11	8	ST11	Bovine	USA	04/09/2007
6612-065	RT002	grp 1	1	1	2	6	1	5	1	ST08	Human	UK	
6612-590	RT015	grp 1	2	5	2	1	1	3	1	ST44	Human	UK	
6612-647	NT	grp 1	1	1	6	6	1	12	12	ST55	Human	UK	
6612-820	RT094	grp 1	1	1	6	4	3	5	1	ST12	Human	UK	
6612-978	RT094	grp 1	1	1	6	4	3	5	1	ST12	Human	UK	
6613-373	RT002	grp 1	1	1	2	6	1	5	1	ST08	Human	UK	
6613-833	RT087	grp 1	4	1	6	1	1	10	1	ST46	Human	UK	
AI149	RT126	grp 5	**5**	8	5	11	9	11	8	ST11	Kangaroo	Australia	
AI15	RT237	grp 5	**5**	8	5	11	9	11	8	ST11	Porcine	Australia	

AI152	NT	grp 5	**5**	8	5	11	9	11	8	ST11	Porcine	Australia	
AI18	RT014	grp 1	**5**	**1**	6	1	5	3	1	New (ST132)	Porcine	Australia	
AI24	RT237	grp 5	**5**	8	5	11	9	11	8	ST11	Porcine	Australia	
AI35	RT237	grp 5	5	8	5	11	9	11	8	ST11	Porcine	Australia	
B1	NT	grp 1	1	1	7	5	1	3	3	ST63	Human	UK	1978
BI-1	RT027	grp 2	1	1	1	10	1	3	5	ST01	Human	USA	26/02/1988
BI-10	RT027	grp 2	1	1	1	10	1	3	5	ST01	Human	USA	10/08/2001
BI-13	RT027	grp 2	1	1	1	10	1	3	5	ST01	Human	USA	09/09/2004
BI-15	RT027	grp 2	1	1	1	10	1	3	5	ST01	Human	USA	09/09/2004
BI-2	RT027	grp 2	1	1	1	10	1	3	5	ST01	Human	USA	14/01/1991
BI-5	RT027	grp 2	1	1	1	10	1	3	5	ST01	Human	USA	25/08/1995
BI-6	RT027	grp 2	1	1	1	10	1	3	5	ST01	Human	USA	20/05/2003
BI-6p	RT027	grp 2	1	1	1	10	1	3	5	ST01	Human	USA	09/09/2004
BI-7	RT027	grp 2	1	1	1	10	1	3	5	ST01	Human	USA	20/05/2003
BI-8	RT027	grp 2	1	1	1	10	1	3	5	ST01	Human	USA	22/01/2004
BI-9	RT001	grp 1	1	1	2	1	1	1	1	ST03	Human	USA	
CD DE2	NT	grp 1	1	1	2	1	1	1	1	ST03			
CD#101	NT	grp 1	1	1	7	5	1	3	3	ST63			
CD#17	NT	grp 4	3	7	3	8	6	9	11	ST37			
CD#371	NT	grp 1	1	1	2	1	1	1	1	ST03			
CD1	RT027	grp 2	1	1	1	10	1	3	5	ST01	Human	UK	12.07.07
CD1010	RT274	grp 1	1	1	7	1	1	3	3	New (ST133)	Human	UK	20/01/2010
CD1049	RT013	grp 1	4	1	6	1	1	5	1	ST45	Human	UK	03/03/2010
CD1061	RT283	grp 1	1	3	2	15	1	3	3	New (ST134)	Human	UK	19/03/2010
CD1075	RT070	grp 1	1	1	6	6	1	12	12	ST55	Human	UK	04/03/2010
CD1077	RT106	grp 1	1	1	2	8	1	7	1	New (ST135)	Human	UK	29/03/2010
CD1079	RT078	grp 5	5	8	5	11	9	11	8	ST11	Human	USA	27/03/2010
CD1080	RT052	grp 1	2	1	6	16	1	5	13	New (ST136)	Human	UK	27/03/2010
CD1099	RT059	grp 1	1	2	2	1	1	5	1	ST53	Human	UK	02/04/2010
CD11	RT027	grp 2	**1**	1	1	10	1	3	5	ST01	Human	UK	21.05.07
CD1108	RT097	grp 1	2	2	2	1	1	1	3	ST21	Human	UK	18/04/2010
CD1128	RT118	grp 1	1	1	2	1	1	7	1	ST42	Human	UK	04/05/2010
CD1132	RT216	grp 1	1	1	2	1	6	5	3	ST33	Human	UK	07/05/2010
CD1141	RT020	grp 1	1	1	2	1	5	3	1	ST02	Human	UK	17/05/2010
CD1143	RT140	grp 1	1	1	6	1	4	3	4	ST26	Human	UK	04/05/2010
CD1144	RT003	grp 1	1	1	6	4	3	5	13	ST57	Human	UK	17/05/2010
CD1149	RT186	grp 1	1	1	2	6	1	7	6	ST51	Human	UK	23/05/2010
CD1153	RT139	grp 1	1	1	2	16	1	12	1	ST52	Human	UK	27/05/2010
CD1157	RT046	grp 1	2	5	8	1	1	3	6	ST35	Human	UK	24/05/2010
CD1165	RT053	grp 1	1	1	7	5	1	3	3	ST63	Human	UK	28/05/2010
CD1170	RT018	grp 1	1	1	2	1	1	5	3	ST17	Human	UK	22/05/2010
CD1171	RT029	grp 1	1	1	2	3	1	3	1	New (ST137)	Human	UK	26/05/2010

CD1199	RT021	grp 1	1	3	6	3	1	5	1	ST56	Human	UK	09/06/2010
CD12	RT027	grp 2	1	1	1	10	1	3	5	ST01	Human	UK	06.07.07
CD1201	RT015	grp 1	2	1	2	1	1	3	1	ST10	Human	UK	11/06/2010
CD1202	RT012	grp 1	1	4	7	1	1	3	3	ST54	Human	UK	09/05/2010
CD1210	RT054	grp 1	1	7	6	1	1	5	6	ST43	Human	UK	18/06/2010
CD1214	RT011	grp 1	2	1	2	16	1	5	1	New (ST138)	Human	UK	04/12/2009
CD1220	RT005	grp 1	2	1	6	1	1	5	1	ST06	Human	UK	20/06/2010
CD1224	RT107	grp 1	2	1	2	1	1	1	3	New (ST139)	Human	UK	14.06.10
CD20	RT027	grp 2	1	1	1	10	1	3	5	ST01	Human	UK	07.08.07
CD25	RT027	grp 2	**1**	1	1	10	1	3	5	ST01	Human	UK	31.07.07
CD305	RT023	grp 3	1	1	4	7	2	8	7	ST22	Human	UK	24.04.08
CD453	RT023	grp 3	**1**	**6**	4	7	2	8	7	ST05	Human	UK	30.09.08
CD527	RT030	grp 1	1	1	2	1	1	5	1	ST48	Human	UK	06/01/2009
CD586	RT017	grp 4	3	7	3	8	6	9	11	ST37	Human	UK	11.03.09
CD59	RT027	grp 2	**1**	1	1	10	1	3	5	ST01	Human	UK	11.08.07
CD60	RT027	grp 2	1	1	1	10	1	3	5	ST01	Human	UK	04.10.07
CD630	RT176	grp 2	1	1	1	10	1	3	5	ST01	Human	UK	04.04.09
CD637	RT017	grp 4	3	7	3	8	6	9	11	ST37	Human	UK	16.04.09
CD679	RT027	grp 2	1	1	1	10	1	3	5	ST01	Human	UK	16.04.09
CD682	RT027	grp 2	1	1	1	10	1	3	5	ST01	Human	UK	27.04.09
CD683	RT027	grp 2	1	1	1	10	1	3	5	ST01	Human	UK	23.04.09
CD689	RT064	grp 1	1	1	2	1	6	5	3	ST33	Human	UK	11/05/2009
CD714	RT050	grp 1	1	1	2	6	1	3	1	ST16	Human	UK	12.06.09
CD718	RT085	grp 4	3	7	10	8	7	2	10	ST39	Human	UK	08/06/2009
CD735	RT081	grp 1	2	1	2	1	1	1	3	New (ST139)	Human	UK	03/07/2009
CD742	RT243	grp 1	2	1	2	1	1	1	3	New (ST139)	Human	UK	01/01/2009
CD759	RT050	grp 1	2	1	6	1	1	5	1	ST06	Human	UK	15.07.09
CD762	RT111	grp 2	1	1	9	9	1	1	2	New (ST140)	Human	UK	31/07/2009
CD767	RT015	grp 1	2	1	2	1	1	3	1	ST10	Human	UK	27.07.09
CD790	RT027	grp 2	1	1	1	10	1	3	5	ST01	Human	UK	02.08.09
CD806	RT027	grp 2	1	1	1	10	1	3	5	ST01	Human	UK	02.09.09
CD81	RT027	grp 2	**1**	**1**	1	10	1	3	5	ST01	Human	UK	28.08.07
CD816	RT017	grp 4	3	7	3	8	6	9	11	ST37	Human	UK	28.09.09
CD825	RT017	grp 4	3	7	3	8	6	9	11	ST37	Human	UK	01.10.09
CD839	RT017	grp 4	3	7	3	8	6	9	11	ST37	Human	UK	29.10.09
CD853	RT062	grp 1	2	5	2	1	1	3	1	ST44	Human	UK	21/10/2009
CD871	RT259	grp 1	1	3	7	1	3	1	6	New (ST141)	Human	UK	06/11/2009
CD877	RT135	grp 2	1	1	9	9	1	3	2	ST41	Human	UK	22/11/2009
CD886	RT116	grp 1	2	1	2	1	1	3	1	ST10	Human	UK	06/12/2009
CD909	RT264	grp 4	8	7	14	8	6	25	15	New (ST142)	Human	UK	17/12/2009
CD914	RT010	grp 1	1	1	6	1	8	5	1	ST15	Human	UK	14/12/2009
CD915	RT126	grp 5	5	8	5	11	9	11	8	ST11	Human	UK	02/12/2009

CD917	RT022	grp 1	1	1	2	6	1	5	3	ST66	Human	UK	20/12/2009
CD955	RT268	grp 1	1	1	2	1	1	1	1	ST03	Human	UK	15/01/2010
CD959	RT262	grp 1	1	11	6	16	1	1	1	New (ST143)	Human	UK	13/01/2010
CD966	RT196	grp 1	1	1	2	2	1	5	3	New (ST144)	Human	UK	22/01/2010
CD970	RT271	grp 1	2	1	6	1	1	5	1	ST06	Human	UK	23/01/2010
CD973	RT002	grp 1	1	1	2	6	1	5	1	ST08	Human	UK	24/11/2009
CDT134	RT002	grp 1	**1**	1	2	6	1	5	1	ST08	Human	UK	
CF3	RT017	grp 4	3	7	3	8	6	9	11	ST37	Human	Belgium	1995
CF5	RT017	grp 4	3	7	3	8	6	19	11	ST86	Human	Belgium	1995
DS209/06	NT	grp 2	1	1	1	10	1	3	5	ST01		UK	
E327 -98	RT126	grp 5	5	8	5	11	9	11	8	ST11	Equine	Switzerland	before 2004
ES173	RT017	grp 4	**3**	7	3	8	6	9	11	ST37	Human	Australia	18/12/2006
ES130	RT280	grp 5	**5**	8	5	11	9	11	8	ST11	Human		
ES166	RT281	grp 5	**5**	**8**	5	11	9	11	8	ST11	Human	Australia	28/06/1905
ES67	RT014	grp 1	**1**	1	6	1	5	3	1	ST13		Australia	
ES84	RT027	grp 2	**1**	**1**	1	10	1	3	5	ST01		Canada	
J9	NT	grp 1	1	1	2	1	1	1	1	ST03	Human	USA	
JGS 6047	NT	grp 1	1	1	2	1	1	1	1	ST03	Equine	USA	
JGS 679	RT078	grp 5	5	8	5	11	9	11	8	ST11	Bovine	USA	
JGS344	NT	grp 1	2	5	8	1	1	3	6	ST35	Murine	USA	
JGS355	RT002	grp 1	2	5	8	1	1	3	6	ST35	Murine	USA	
JGS356	RT002	grp 1	2	5	8	1	1	3	6	ST35	Murine	USA	
JGS357	RT002	grp 1	2	5	8	1	1	3	6	ST35	Murine	USA	
JGS360	RT002	grp 1	2	5	8	1	1	3	6	ST35	Murine	USA	
JGS6042	RT002	grp 1	1	1	2	6	1	5	1	ST08	Equine	USA	
JGS6050	RT020	grp 1	1	1	2	1	5	3	1	ST02	Canine	USA	
JGS655	NT	grp 1	1	1	2	6	1	5	1	ST08	Porcine	USA	
JGS673	RT078	grp 5	5	8	5	11	9	11	8	ST11	Bovine	USA	
JGS674	RT078	grp 5	5	8	5	11	9	11	8	ST11	Bovine	USA	
JGS675	RT078	grp 5	5	8	5	11	9	11	8	ST11	Bovine	USA	
JGS676	RT078	grp 5	5	8	5	11	9	11	8	ST11	Bovine	USA	
JGS677	RT078	grp 5	5	8	5	11	9	11	8	ST11	Bovine	USA	
JGS688	RT126	grp 5	5	8	5	11	9	11	8	ST11	Porcine	USA	
JGS691	RT078	grp 5	5	8	5	11	9	11	8	ST11	Porcine	USA	
JGS692	RT002	grp 1	1	1	2	1	1	5	1	ST48	Porcine	USA	
M1	NT	grp 1	1	1	6	1	1	13	1	ST64			
M120	RT078	grp 5	5	8	5	11	9	11	8	ST11	Human	Ireland	
M13	NT	grp 1	1	1	6	1	8	5	1	ST15	Human		
M68	RT017	grp 4	3	7	3	8	6	9	11	ST37	Human	Ireland	2006
metal 1	NT	grp 5	5	8	5	11	9	11	8	ST11	Bovine	USA	
O1-027	RT027	grp 2	**1**	1	1	10	1	3	5	ST01	Human	UK	
O1-078	RT078	grp 5	**5**	8	5	11	9	11	8	ST11	Human	UK	

PMH13	RT010	grp 1	**1**	1	6	1	8	5	1	ST15	Human	Australia	09/01/2007
PMH44	RT126	grp 5	**5**	8	5	11	9	11	8	ST11	Human	Australia	16/08/2008
R10287	RT027	grp 2	1	1	1	10	1	3	5	ST01	Human	France	
R1040	RT212	grp 3	**1**	6	4	7	2	8	7	ST05	Human		
R10459	RT106	grp 1	1	1	2	1	1	7	1	ST42	Human		
R20291	RT027	grp 2	1	1	1	10	1	3	5	ST01	Human	UK	2006
R20352	RT027	grp 2	1	1	1	10	1	3	5	ST01	Human	Canada	2005
R279	NT	grp 3	**1**	**6**	4	7	2	8	7	ST05	Human		
R711	RT031	grp 1	1	1	2	16	1	3	1	ST29	Human		
R714	RT001	grp 1	**1**	1	2	1	1	1	1	ST03	Human		
R8366	RT001	grp 1	1	1	2	1	1	1	1	ST03	Human		
R839	NT	grp 1	1	**1**	2	1	1	1	1	ST03	Human		
R894	RT002	grp 1	**1**	1	2	6	1	5	1	ST08	Human		
RPH101	NT	grp 5	5	8	5	11	9	11	8	ST11	Human	Australia	27/01/2007
RPH13	NT	grp 1	1	1	7	5	1	3	3	ST63	Human	Australia	24/08/2006
RPH35	RT087	grp 1	**4**	1	6	1	1	10	12	New (ST145)	Human	Australia	30/08/2006
RPH56	RT014	grp 1	**1**	**1**	2	1	5	3	1	ST02	Human	Australia	02/06/2006
RPH61	RT005	grp 1	**2**	1	6	1	1	5	1	ST06	Human	Australia	04/11/2006
RT023	RT023	grp 1	1	6	4	7	2	8	7	ST05			
RT026	RT026	grp 1	1	1	7	1	1	5	1	ST07			
RT042	RT042	grp 1	2	1	6	1	1	5	1	ST06			
RT050	RT050	grp 1	1	1	2	5	1	3	1	ST18			
RT176	RT176	grp 2	1	1	1	10	1	3	5	ST01			
S10.1014	RT027	grp 2	1	1	1	10	1	3	5	ST01	Human	UK	2010
S10.1486	NT	grp 1	**1**	**1**	6	1	5	3	1	ST13	Human	UK	2010
S10.1920	RT002	grp 1	1	1	2	6	1	5	4	New (ST146)	Human	UK	2010
S10.358	RT078	grp 5	**5**	**8**	5	11	9	11	8	ST11	Human	UK	2010
S10.564	RT027	grp 2	**1**	1	1	10	1	3	5	ST01	Human	UK	2010
slaughter 1	RT078	grp 5	5	8	5	11	9	11	8	ST11	Bovine	USA	
T7	NT	grp 1	1	1	2	1	1	1	1	ST03	Human		
VPI 10463/ATCC 43255	RT087	grp 1	4	1	6	1	1	10	1	ST46	Human	USA	1935
WA 107	RT127	grp 5	**5**	8	5	11	9	11	8	ST11	Human	Australia	01/03/2006
WA 12	RT239	grp 5	10	8	5	11	9	11	8	New (ST147)	Human	Australia	
WA 122	RT002	grp 1	**1**	1	2	6	1	5	1	ST08	Human	Australia	14/04/2006
WA 13	RT291	grp 5	**5**	8	5	11	9	11	20	New (ST148)	Human	Australia	31/12/2005
WA 146	NT	grp 1	**1**	1	6	6	1	12	12	ST55	Human	Australia	27/12/2005
WA 15	RT002	grp 1	**1**	**1**	2	6	1	5	1	ST08	Human	Australia	05/01/2009
WA 151	RT237	grp 5	**5**	8	5	11	9	11	8	ST11	Human	Australia	11/07/2006
WA 156	RT012	grp 1	**1**	4	7	1	1	3	3	ST54	Human	Australia	14/06/2006
WA 158	NT	grp 1	**1**	**5**	7	1	1	3	1	ST34	Human	Australia	08/06/2006
WA 161	RT010	grp 1	**1**	**1**	6	1	8	5	1	ST15	Human	Australia	05/07/2006
WA 169	RT081	grp 1	**1**	1	6	1	1	6	1	ST09	Human	Australia	02/06/2006

WA 48	RT127	grp 5	**5**	8	5	11	9	11	8	ST11	Human	Australia	14/11/2005
WA 52	RT014	grp 1	**1**	1	2	1	5	3	1	ST02	Human	Australia	02/09/2005
WA 68	RT009	grp 1	**1**	1	2	1	1	1	1	ST03	Human	Australia	09/10/2005
WA 76	RT001	grp 1	**1**	1	2	1	1	1	1	ST03	Human	Australia	13/10/2005
WA 77	RT127	grp 5	**5**	**8**	5	11	9	11	8	ST11	Human	Australia	14/10/2005
WA 80	NT	grp 1	**1**	**1**	6	6	1	12	12	ST55	Human	Australia	19/10/2005
WA 93	RT054	grp 1	**1**	7	6	1	1	5	6	ST43	Human	Australia	28/01/2006
WA 94	RT078	grp 5	**5**	8	5	11	9	11	8	ST11	Human	Australia	19/01/2006
