# Supplementary material for: Macro and Micro Diversity of Clostridium difficile Isolates from Diverse Sources and Geographical Locations
Source: PLoS One. 2012 Mar 2;7(3):e31559. doi: 10.1371/journal.pone.0031559 (PMC3292544; doi:10.1371/journal.pone.0031559)
Supplement: Data S2 — Strains in PCR ribotype 027 depth study. (PDF) [file pone.0031559.s010.pdf]

Strain	Ribotype	atpA	dxr	glyA	recA	sodA	tpi	ST		Origin	Date isolated
2053	RT027	1	1	10	1	3	5	ST01			
5359	NT	1	1	10	1	3	5	ST01			
5370	RT027	1	1	10	1	3	5	ST01	Bovine	USA	26/06/2007
5373	RT027	1	1	10	1	3	5	ST01	Bovine	USA	20/03/2007
5427	RT027	1	1	10	1	3	5	ST01	Bovine	USA	31/07/2007
6014	RT027	1	1	10	1	3	5	ST01	Bovine	USA	
2004101	RT027	1	1	10	1	3	5	ST01	Human	USA	2004
2004102	RT027	1	1	10	1	3	5	ST01	Human	USA	2004
2004118	RT027	1	1	10	1	3	5	ST01	Human	USA	2004
2004163	RT027	1	1	10	1	3	5	ST01	Human	USA	2004
2005079	NT	1	1	10	1	3	5	ST01	Human	USA	2005
2006237	RT027	1	1	10	1	3	5	ST01	Equine	USA	2006
2006439	RT027	1	1	10	1	3	5	ST01	Food	USA	2006
2007042	RT027	1	1	10	1	3	5	ST01	Food	USA	2007
2007140	RT027	1	1	10	1	3	5	ST01	Human	USA	2007
2007218	RT027	1	1	10	1	3	5	ST01	Food	USA	2007
2007222	RT027	1	1	10	1	3	5	ST01	Food	USA	2007
2007223	RT027	1	1	10	1	3	5	ST01	Food	USA	2007
2007235	NT	1	1	10	1	3	5	ST01	Food	USA	2007
2007825	RT027	1	1	10	1	3	5	ST01	Human	USA	2007
2007828	NT	1	1	10	1	3	5	ST01	Human	USA	2007
2007830	RT027	1	1	10	1	3	5	ST01	Human	USA	2007
2007832	RT027	1	1	10	1	3	5	ST01	Human	USA	2007
2007833	RT027	1	1	10	1	3	5	ST01	Human	USA	2007
2007839	NT	1	1	10	1	3	5	ST01	Human	USA	2007
2007850	RT027	1	1	10	1	3	5	ST01	Household	USA	2007
2007855	RT027	1	1	10	1	3	5	ST01	Bovine	USA	2007
5354 (17/4)	RT027	1	1	10	1	3	5	ST01	Bovine	USA	
BI-1	RT027	1	1	10	1	3	5	ST01	Human	USA	26/02/1988
BI-10	RT027	1	1	10	1	3	5	ST01	Human	USA	10/08/2001
BI-13	RT027	1	1	10	1	3	5	ST01	Human	USA	09/09/2004
BI-15	RT027	1	1	10	1	3	5	ST01	Human	USA	09/09/2004
BI-2	RT027	1	1	10	1	3	5	ST01	Human	USA	14/01/1991
BI-5	RT027	1	1	10	1	3	5	ST01	Human	USA	25/08/1995
BI-6	RT027	1	1	10	1	3	5	ST01	Human	USA	20/05/2003
BI-6p	RT027	1	1	10	1	3	5	ST01	Human	USA	09/09/2004
BI-7	RT027	1	1	10	1	3	5	ST01	Human	USA	20/05/2003
BI-8	RT027	1	1	10	1	3	5	ST01	Human	USA	22/01/2004
CD1	RT027	1	1	10	1	3	5	ST01	Human	UK	12.07.07
CD11	RT027	1	1	10	1	3	5	ST01	Human	UK	21.05.07
CD12	RT027	1	1	10	1	3	5	ST01	Human	UK	06.07.07
CD20	RT027	1	1	10	1	3	5	ST01	Human	UK	07.08.07
CD25	RT027	1	1	10	1	3	5	ST01	Human	UK	31.07.07
CD59	RT027	1	1	10	1	3	5	ST01	Human	UK	11.08.07
CD60	RT027	1	1	10	1	3	5	ST01	Human	UK	04.10.07
CD630	RT176	1	1	10	1	3	5	ST01	Human	UK	04.04.09
CD679	RT027	1	1	10	1	3	5	ST01	Human	UK	16.04.09
CD682	RT027	1	1	10	1	3	5	ST01	Human	UK	27.04.09
CD683	RT027	1	1	10	1	3	5	ST01	Human	UK	23.04.09
CD790	RT027	1	1	10	1	3	5	ST01	Human	UK	02.08.09
CD806	RT027	1	1	10	1	3	5	ST01	Human	UK	02.09.09
CD81	RT027	1	1	10	1	3	5	ST01	Human	UK	28.08.07
DS209/06	NT	1	1	10	1	3	5	ST01		UK	
ES84	RT027	1	1	10	1	3	5	ST01		Australia	
O1-027	RT027	1	1	10	1	3	5	ST01	Human	UK	
R10287	RT027	1	1	10	1	3	5	ST01	Human	France	
R20291	RT027	1	1	10	1	3	5	ST01	Human	UK	2006
R20352	RT027	1	1	10	1	3	5	ST01	Human	Canada	2005
RT176	RT176	1	1	10	1	3	5	ST01			
S10.1014	RT027	1	1	10	1	3	5	ST01	Human	UK	2010
S10.564	RT027	1	1	10	1	3	5	ST01	Human	UK	2010
2007098	NT	1	11	1	1	3	2	ST32	Human	USA	2007

2007195	NT	1	9	9	1	3	2	ST41	Human	USA	2007
CD877	RT135	1	9	9	1	3	2	ST41	Human	UK	22/11/2009
2007826	NT	5	11	1	1	3	2	ST61	Human	USA	2007
2007843	NT	5	11	1	1	3	2	ST61	Food	USA	2007
2007844	NT	5	11	1	1	3	2	ST61	Food	USA	2007
8864	RT036	1	1	9	1	3	1	ST62	Human	UK	before 2004
2007827	RT262	1	9	9	1	3	5	ST67	Human	USA	2007
2007829	RT262	1	9	9	1	3	5	ST67	Human	USA	2007
CD762	RT111	1	9	9	1	1	2	New (ST140)	Human	UK	31/07/2009
