# Supplementary material for: Macro and Micro Diversity of Clostridium difficile Isolates from Diverse Sources and Geographical Locations
Source: PLoS One. 2012 Mar 2;7(3):e31559. doi: 10.1371/journal.pone.0031559 (PMC3292544; doi:10.1371/journal.pone.0031559)
Supplement: Data S3 — Strains in PCR ribotype 078 depth study. (PDF) [file pone.0031559.s011.pdf]

Strain	Ribotype	adk	atpA	dxr	glyA	recA	sodA	tpi	ST	Origin	Location	Date isolated
IS58	RT033	5	8	5	11	9	11	8	ST11	Human	Australia	before 2004
5350	RT078	5	8	5	11	9	11	8	ST11	Bovine	USA	
5361	RT078	5	8	5	11	9	11	8	ST11	Bovine	USA	26/12/2006
5363	RT078	5	8	5	11	9	11	8	ST11	Bovine	USA	20/03/2007
5384	RT078	5	8	5	11	9	11	8	ST11	Bovine	USA	20/03/2007
5397	RT078	5	8	5	11	9	11	8	ST11	Bovine	USA	26/06/2007
5407	RT078	5	8	5	11	9	11	8	ST11	Bovine	USA	26/09/2006
5408	RT078	5	8	5	11	9	11	8	ST11	Bovine	USA	26/06/2007
5416	RT078	5	8	5	11	9	11	8	ST11	Bovine	USA	2006
5428	RT078	5	8	5	11	9	11	8	ST11	Bovine	USA	20/03/2007
5429	RT078	5	8	5	11	9	11	8	ST11	Bovine	USA	15/10/2007
5432	RT078	5	8	5	11	9	11	8	ST11	Bovine	USA	23/01/2007
5444	RT078	5	8	5	11	9	11	8	ST11	Bovine	USA	15/10/2007
5898	RT078	5	8	5	11	9	11	8	ST11	Bovine	USA	20/03/2007
5904	RT078	5	8	5	11	9	11	8	ST11	Bovine	USA	15/10/2007
5911	RT078	5	8	5	11	9	11	8	ST11	Bovine	USA	17/04/2007
5917	RT078	5	8	5	11	9	11	8	ST11	Bovine	USA	15/10/2007
5921	RT078	5	8	5	11	9	11	8	ST11	Bovine	USA	20/11/2006
5938	RT078	5	8	5	11	9	11	8	ST11	Bovine	USA	20/11/2006
5946	RT078	5	8	5	11	9	11	8	ST11	Bovine	USA	20/03/2007
5963	RT078	5	8	5	11	9	11	8	ST11	Bovine	USA	20/11/2006
5968	RT078	5	8	5	11	9	11	8	ST11	Bovine	USA	20/03/2007
5982	RT078	5	8	5	11	9	11	8	ST11	Bovine	USA	20/11/2006
5983	RT078	5	8	5	11	9	11	8	ST11	Bovine	USA	20/03/2007
5984	RT078	5	8	5	11	9	11	8	ST11	Bovine	USA	15/10/2007
5986	RT078	5	8	5	11	9	11	8	ST11	Bovine	USA	31/07/2007
5987	RT078	5	8	5	11	9	11	8	ST11	Bovine	USA	15/10/2007
5996	RT078	5	8	5	11	9	11	8	ST11	Bovine	USA	15/10/2007
6005	RT078	5	8	5	11	9	11	8	ST11	Bovine	USA	26/06/2007
6007	RT078	5	8	5	11	9	11	8	ST11	Bovine	USA	2007
6007	RT078	5	8	5	11	9	11	8	ST11	Bovine	USA	2007
6015	RT078	5	8	5	11	9	11	8	ST11	Bovine	USA	15/10/2007
6021	RT078	5	8	5	11	9	11	8	ST11	Bovine	USA	20/03/2007
10/33	RT078	5	8	5	11	9	11	8	ST11			
13/03	RT078	5	8	5	11	9	11	8	ST11			
19/07	RT078	5	8	5	11	9	11	8	ST11			
19/09	RT078	5	8	5	11	9	11	8	ST11			
17/10	RT078	5	8	5	11	9	11	8	ST11			
3/11	RT078	5	8	5	11	9	11	8	ST11			
80249	RT078	5	8	5	11	9	11	8	ST11			20/03/2007
2005325	RT078	5	8	5	11	9	11	8	ST11	Human	USA	2005
2005511	RT078	5	8	5	11	9	11	8	ST11	Porcine	USA	2005
2005515	RT078	5	8	5	11	9	11	8	ST11	Porcine	USA	2005
2006238	RT078	5	8	5	11	9	11	8	ST11	Bovine	USA	2006
2006239	RT078	5	8	5	11	9	11	8	ST11	Bovine	USA	2006
2006240	RT078	5	8	5	11	9	11	8	ST11	Bovine	USA	2006
2006241	RT078	5	8	5	11	9	11	8	ST11	Bovine	USA	2006
2006243	RT078	5	8	5	11	9	11	8	ST11	Bovine	USA	2006
2006244	RT078	5	8	5	11	9	11	8	ST11	Bovine	USA	2006
2006245	RT078	5	8	5	11	9	11	8	ST11	Bovine	USA	2006
2006246	RT078	5	8	5	11	9	11	8	ST11	Bovine	USA	2006
2006253	RT078	5	8	5	11	9	11	8	ST11	Bovine	USA	2006
2006254	RT078	5	8	5	11	9	11	8	ST11	Bovine	USA	2006
2006354	RT078	5	8	5	11	9	11	8	ST11	Human	USA	2006
2006379	RT078	5	8	5	11	9	11	8	ST11	Human	USA	2006
2006437	RT078	5	8	5	11	9	11	8	ST11	Food	USA	2006
2006438	RT078	5	8	5	11	9	11	8	ST11	Food	USA	2006
2006460	RT078	5	8	5	11	9	11	8	ST11	Human	USA	2006
2007007	RT078	5	8	5	11	9	11	8	ST11	Human	USA	2007
2007011	RT078	5	8	5	11	9	11	8	ST11	Human	USA	2007
2007024	RT078	5	8	5	11	9	11	8	ST11	Human	USA	2007
2007054	RT078	5	8	5	11	9	11	8	ST11	Human	USA	2007

2007219	RT078	5	8	5	11	9	11	8	ST11	Food	USA	2007
2007230	RT078	5	8	5	11	9	11	8	ST11	Food	USA	2007
2007334	RT078	5	8	5	11	9	11	8	ST11	Human	USA	2007
2007361	RT078	5	8	5	11	9	11	8	ST11	Human	USA	2007
2007380	RT078	5	8	5	11	9	11	8	ST11	Human	USA	2007
2007606	RT078	5	8	5	11	9	11	8	ST11	Food	USA	2007
2007607	RT078	5	8	5	11	9	11	8	ST11	Food	USA	2007
2007786	RT078	5	8	5	11	9	11	8	ST11	Human	Italy	2007
2007792	RT078	5	8	5	11	9	11	8	ST11	Human	Italy	2007
2007834	RT078	5	8	5	11	9	11	8	ST11	Human	USA	2007
2007835	RT078	5	8	5	11	9	11	8	ST11	Human	USA	2007
2007838	RT078	5	8	5	11	9	11	8	ST11	Human	USA	2007
6600639	RT078	**5**	8	5	11	9	11	8	ST11	Human	UK	
6614376	RT078	**5**	**8**	5	11	9	11	8	ST11	Human	UK	
6616023	RT078	**5**	**8**	5	11	9	11	8	ST11	Human	UK	
6616104	RT078	**5**	8	5	11	9	11	8	ST11	Human	UK	
078W	RT078	5	8	5	11	9	11	8	ST11			
17/50	RT078	5	8	5	11	9	11	8	ST11			
18/21	RT078	5	8	5	11	9	11	8	ST11			
19/44	RT078	5	8	5	11	9	11	8	ST11			
19/52	RT078	5	8	5	11	9	11	8	ST11			
19/72	RT078	5	8	5	11	9	11	8	ST11			
20/28	RT078	5	8	5	11	9	11	8	ST11			
22/31	RT078	5	8	5	11	9	11	8	ST11			
23/41	RT078	5	8	5	11	9	11	8	ST11			
25/40	RT078	5	8	5	11	9	11	8	ST11			
5353 (3/20)	RT078	5	8	5	11	9	11	8	ST11	Bovine	USA	20/03/2007
5379 (26/12)	RT078	5	8	5	11	9	11	8	ST11	Bovine	USA	
5404 (17/4)	RT078	5	8	5	11	9	11	8	ST11	Bovine	USA	17/04/2007
5404 (9/26)	RT078	5	8	5	11	9	11	8	ST11	Bovine	USA	26/09/2006
5424 (2/20)	RT078	5	8	5	11	9	11	8	ST11	Bovine	USA	20/02/2007
5964 (20/11)	RT078	5	8	5	11	9	11	8	ST11	Bovine	USA	20/11/2006
5964 (9/1)	RT078	5	8	5	11	9	11	8	ST11	Bovine	USA	09/01/2007
CD1079	RT078	5	8	5	11	9	11	8	ST11	Human	USA	27/03/2010
JGS 679	RT078	5	8	5	11	9	11	8	ST11	Bovine	USA	
JGS673	RT078	5	8	5	11	9	11	8	ST11	Bovine	USA	
JGS674	RT078	5	8	5	11	9	11	8	ST11	Bovine	USA	
JGS675	RT078	5	8	5	11	9	11	8	ST11	Bovine	USA	
JGS676	RT078	5	8	5	11	9	11	8	ST11	Bovine	USA	
JGS677	RT078	5	8	5	11	9	11	8	ST11	Bovine	USA	
JGS691	RT078	5	8	5	11	9	11	8	ST11	Porcine	USA	
M120	RT078	5	8	5	11	9	11	8	ST11	Human	Ireland	
O1-078	RT078	**5**	8	5	11	9	11	8	ST11	Human	UK	
S10.358	RT078	**5**	**8**	5	11	9	11	8	ST11	Human	UK	2010
slaughter 1	RT078	5	8	5	11	9	11	8	ST11	Bovine	USA	
WA 94	RT078	**5**	8	5	11	9	11	8	ST11	Human	Australia	19/01/2006
2005093	RT126	5	8	5	11	9	11	8	ST11	Porcine	USA	2005
2007019	RT126	5	8	5	11	9	11	8	ST11	Human	USA	2007
2007229	RT126	5	8	5	11	9	11	8	ST11	Food	USA	2007
2007600	RT126	5	8	5	11	9	11	8	ST11	Human	Spain	2007
2007601	RT126	5	8	5	11	9	11	8	ST11	Human	Spain	2007
AI149	RT126	**5**	8	5	11	9	11	8	ST11	Kangaroo	Australia	
CD915	RT126	5	8	5	11	9	11	8	ST11	Human	UK	02/12/2009
E327 -98	RT126	5	8	5	11	9	11	8	ST11	Equine	Switzerland	before 2004
JGS688	RT126	5	8	5	11	9	11	8	ST11	Porcine	USA	
PMH44	RT126	**5**	8	5	11	9	11	8	ST11	Human	Australia	16/08/2008
WA 107	RT127	**5**	8	5	11	9	11	8	ST11	Human	Australia	01/03/2006
WA 48	RT127	**5**	8	5	11	9	11	8	ST11	Human	Australia	14/11/2005
WA 48	RT127	**5**	8	5	11	9	11	8	ST11	Human	Australia	14/11/2005
WA 77	RT127	**5**	**8**	5	11	9	11	8	ST11	Human	Australia	14/10/2005
WA 77	RT127	**5**	**8**	5	11	9	11	8	ST11	Human	Australia	14/10/2005
AI15	RT237	**5**	8	5	11	9	11	8	ST11	Porcine	Australia	
AI24	RT237	**5**	8	5	11	9	11	8	ST11	Porcine	Australia	

AI35	RT237	5	8	5	11	9	11	8	ST11	Porcine	Australia	
WA 151	RT237	**5**	8	5	11	9	11	8	ST11	Human	Australia	11/07/2006
ES130	RT280	**5**	8	5	11	9	11	8	ST11	Human		
ES166	RT281	**5**	**8**	5	11	9	11	8	ST11	Human	Australia	28/06/1905
2054	NT	5	**8**	5	11	9	11	8	ST11			
5342	NT	5	8	5	11	9	11	8	ST11	Bovine	USA	
5379	NT	5	8	5	11	9	11	8	ST11	Bovine	USA	
5416	NT	5	8	5	11	9	11	8	ST11	Bovine	USA	2006
5468	NT	5	8	5	11	9	11	8	ST11	Bovine	USA	
5912	NT	5	8	5	11	9	11	8	ST11	Bovine	USA	
5927	NT	5	8	5	11	9	11	8	ST11	Bovine	USA	
5927	NT	5	8	5	11	9	11	8	ST11	Bovine	USA	
5933	NT	5	8	5	11	9	11	8	ST11	Bovine	USA	
5954	NT	5	8	5	11	9	11	8	ST11	Bovine	USA	
5992	NT	5	8	5	11	9	11	8	ST11	Bovine	USA	
6004	NT	5	8	5	11	9	11	8	ST11	Bovine	USA	
6065	NT	5	8	5	11	9	11	8	ST11	Bovine	USA	
6067	NT	5	8	5	11	9	11	8	ST11	Bovine	USA	
30256	NT	5	8	5	11	9	11	8	ST11	Bovine	USA	
31807	NT	5	8	5	11	9	11	8	ST11			
2005088	NT	5	8	5	11	9	11	8	ST11	Porcine	USA	2005
2005094	NT	5	8	5	11	9	11	8	ST11	Porcine	USA	2005
2005508	NT	5	8	5	11	9	11	8	ST11	Porcine	USA	2005
2005519	NT	5	8	5	11	9	11	8	ST11	Porcine	USA	2005
2007134	NT	5	8	5	11	9	11	8	ST11	Human	USA	2007
2007206	NT	5	8	5	11	9	11	8	ST11	Human	USA	2007
2007224	NT	5	8	5	11	9	11	8	ST11	Food	USA	2007
5945 (2/21)	NT	5	8	5	11	9	11	8	ST11	Bovine	USA	20/11/2006
5994 (9/4)	NT	5	8	5	11	9	11	8	ST11	Bovine	USA	04/09/2007
AI152	NT	**5**	8	5	11	9	11	8	ST11	Porcine	Australia	
metal 1	NT	5	8	5	11	9	11	8	ST11	Bovine	USA	
RPH101	NT	5	8	5	11	9	11	8	ST11	Human	Australia	27/01/2007
WA 12	RT239	10	8	5	11	9	11	8	New (ST147)	Human	Australia	
WA 13	RT291	**5**	8	5	11	9	11	20	New (ST148)	Human	Australia	31/12/2005
