# Supplementary material for: Macro and Micro Diversity of Clostridium difficile Isolates from Diverse Sources and Geographical Locations
Source: PLoS One. 2012 Mar 2;7(3):e31559. doi: 10.1371/journal.pone.0031559 (PMC3292544; doi:10.1371/journal.pone.0031559)
Supplement: Data S4 — PCR ribotypes associated with RT. Griffiths = data taken from Griffiths et al , Dingle = data taken from Dingle et al , Stabler = data from this study, PCR ribotypes (RT) tested (highlighted indicates previously untested RT), Nos = number of isolates with RT and Sequence Type (ST) combination in this study, ST = sequence type (highlight indicates a new RT/ST association, ‘new’ indicates a novel ST. (PDF) [file pone.0031559.s012.pdf]

Griffiths	Dingle	Stabler	Nos	Con	ST	adk	atpA	dxr	glyA	recA	sodA	tpi	
RT001	RT001	RT001	15	RT001	**3**	1	1	2	1	1	1	1	
		RT002	1	RT002	**2**	1	1	2	1	5	3	1	
RT002	RT002	RT002	10	RT002	**8**	1	1	2	6	1	5	1	
		RT002	4	RT002	**35**	2	5	8	1	1	3	6	
		RT002	1	RT002	**48**	1	1	2	1	1	5	1	
		RT002	1	RT002	New	1	1	2	6	1	5	4	New (ST146)
RT003	RT003			RT003	**12**	1	1	6	4	3	5	1	
	RT003	RT003	1	RT003	**57**	1	1	6	4	3	5	13	
RT005	RT005	RT005	2	RT005	**6**	2	1	6	1	1	5	1	
	RT005			RT005	**72**	2	1	6	13	1	5	1	
	RT005			RT005	**89**	2	1	6	1	1	21	1	
	RT005			RT005	**90**	2	1	16	1	1	5	1	
		RT005	1	RT005	New	2	1	6	8	1	5	1	New (ST131)
RT009	RT009	RT009	1	RT009	**3**	1	1	2	1	1	1	1	
RT010		RT010	3	RT010	**15**	1	1	6	1	8	5	1	
RT011	RT011			RT011	**36**	2	1	2	3	1	5	1	
	RT011			RT011	**77**	2	1	6	1	3	5	1	
		RT011	1	RT011	New	2	1	2	16	1	5	1	New (ST138)
RT012	RT012	RT012	3	RT012	**54**	1	4	7	1	1	3	3	
	RT013	RT013	1	RT013	**45**	4	1	6	1	1	5	1	
	RT013			RT013	**71**	4	1	6	1	1	15	1	
	RT013			RT013	**78**	4	1	6	1	1	1	1	
RT014	RT014	RT014	2	RT014	**2**	1	1	2	1	5	3	1	
		RT014	1	RT014	**13**	1	1	6	1	5	3	1	
RT014	RT014			RT014	**14**	1	1	2	1	5	5	3	
	RT014	RT014	1	RT014	**49**	1	1	2	1	5	3	3	
	RT014			RT014	**50**	1	1	6	1	5	3	17	
		RT014	1	RT014	New	**5**	**1**	6	1	5	3	1	New (ST132)
RT015	RT015	RT015	3	RT015	**10**	2	1	2	1	1	3	1	
	RT015	RT015	4	RT015	**44**	2	5	2	1	1	3	1	
RT017	RT017	RT017	8	RT017	**37**	3	7	3	8	6	9	11	
		RT017	1	RT017	**86**	3	7	3	8	6	19	11	
RT018	RT018	RT018	1	RT018	**17**	1	1	2	1	1	5	3	
RT019	RT019			RT019	**67**	1	1	9	9	1	3	5	
RT020	RT020	RT020	2	RT020	**2**	1	1	2	1	5	3	1	
	RT020			RT020	**28**	1	1	2	1	1	3	1	
	RT020			RT020	**68**	1	4	2	1	5	3	1	
RT021	RT021	RT021	1	RT021	**56**	1	3	6	3	1	5	1	
	RT021			RT021	**70**	1	3	6	3	1	14	1	
	RT022	RT022	1	RT022	**66**	1	1	2	6	1	5	3	
RT023	RT023	RT023	4	RT023	**5**	1	6	4	7	2	8	7	
RT023	RT023	RT023	1	RT023	**22**	1	1	4	7	2	8	7	
	RT023			RT023	**25**	6	6	4	7	2	8	7	
RT026	RT026	RT026	1	RT026	**7**	1	1	7	1	1	5	1	
RT027	RT027	RT027	53	RT027	**1**	1	1	1	10	1	3	5	
		RT029	1	RT029	New	1	1	2	3	1	3	1	New (ST137)
		RT030	1	RT030	**48**	1	1	2	1	1	5	1	
		RT031	1	RT031	**29**	1	1	2	16	1	3	1	
		RT033	1	RT033	**11**	5	8	5	11	9	11	8	

RT035				RT035	**40**	4	5	6	1	3	1	1	
RT036				RT036	**1**	1	1	1	10	1	3	5	
		RT036	1	RT036	**62**	1	1	1	9	1	3	1	
	RT038			RT038	**48**	1	1	2	1	1	5	1	
RT039				RT039	**26**	1	1	6	1	4	3	4	
		RT042	1	RT042	**6**	2	1	6	1	1	5	1	
RT046	RT046	RT046	1	RT046	**35**	2	5	8	1	1	3	6	
		RT050	1	RT050	**6**	2	1	6	1	1	5	1	
RT050	RT050	RT050	1	RT050	**16**	1	1	2	6	1	3	1	
RT050	RT050	RT050	2	RT050	**18**	1	1	2	5	1	3	1	
		RT052	1	RT052	New	2	1	6	16	1	5	13	New (ST136)
	RT053	RT053	1	RT053	**63**	1	1	7	5	1	3	3	
	RT054	RT054	2	RT054	**43**	1	7	6	1	1	5	6	
RT056	RT056			RT056	**34**	1	5	7	1	1	3	1	
	RT056			RT056	**58**	1	5	7	1	1	13	1	
		RT059	1	RT059	**53**	1	2	2	1	1	5	1	
RT060				RT060	**38**	3	7	10	8	6	2	9	
		RT062	1	RT062	**44**	2	5	2	1	1	3	1	
	RT062			RT062	**75**	7	3	13	1	10	1	1	
RT063				RT063	**5**	1	6	4	7	2	8	7	
RT064		RT064	1	RT064	**33**	1	1	2	1	6	5	3	
				RT066	**11**	5	8	5	11	9	11	8	
RT067				RT067	**27**	1	1	6	1	1	3	4	
	RT070			RT070	**15**	1	1	6	1	8	5	1	
	RT070	RT070	1	RT070	**55**	1	1	6	6	1	12	12	
RT072	RT072			RT072	**3**	1	1	2	1	1	1	1	
RT072				RT072	**69**	1	1	7	1	1	1	1	
RT076	RT076			RT076	**2**	1	1	2	1	5	3	1	
RT078	RT078	RT078	106	RT078	**11**	5	8	5	11	9	11	8	
RT081	RT081	RT081	1	RT081	**9**	1	1	6	1	1	6	1	
		RT081	1	RT081	New	2	1	2	1	1	1	3	New (ST139)
RT085		RT085	1	RT085	**39**	3	7	10	8	7	2	10	
		RT087	2	RT087	**46**	4	1	6	1	1	10	1	
		RT087	1	RT087	New	**4**	1	6	1	1	10	12	New (ST145)
		RT094	2	RT094	**12**	1	1	6	4	3	5	1	
	RT097	RT097	1	RT097	**21**	2	2	2	1	1	1	3	
RT103	RT103			RT103	**53**	1	2	2	1	1	5	1	
	RT103			RT103	**73**	1	2	12	1	1	5	1	
	RT103			RT103	**76**	1	1	6	1	1	1	1	
	RT104			RT104	**48**	1	1	2	1	1	5	1	
	RT106			RT106	**41**	1	1	9	9	1	3	2	
RT106	RT106	RT106	9	RT106	**42**	1	1	2	1	1	7	1	
		RT106	1	RT106	New	1	1	2	8	1	7	1	New (ST135)
		RT107	1	RT107	New	2	1	2	1	1	1	3	New (ST139)
RT110	RT110			RT110	**19**	1	1	8	2	1	1	3	
		RT111	1	RT111	New	1	1	9	9	1	1	2	New (ST140)
RT115	RT115			RT115	**3**	1	1	2	1	1	1	1	
		RT116	1	RT116	**10**	2	1	2	1	1	3	1	
RT118		RT118	1	RT118	**42**	1	1	2	1	1	7	1	
		RT126	10	RT126	**11**	5	8	5	11	9	11	8	

		RT127	3	RT127	**11**	5	8	5	11	9	11	8	
RT129	RT129			RT129	**13**	1	1	6	1	5	3	1	
		RT135	1	RT135	**41**	1	1	9	9	1	3	2	
RT137	RT137			RT137	**4**	1	2	2	1	1	5	3	
	RT138			RT138	**23**	3	7	14	14	11	16	15	
	RT139	RT139	1	RT139	**52**	1	1	2	6	1	12	1	
RT140	RT140	RT140	1	RT140	**26**	1	1	6	1	4	3	4	
RT153				RT153	**32**	1	1	11	1	1	3	2	
RT159				RT159	**8**	1	1	2	6	1	5	1	
RT174	RT174			RT174	**42**	1	1	2	1	1	7	1	
		RT176	2	RT176	**1**	1	1	1	10	1	3	5	
RT186	RT186	RT186	1	RT186	**51**	1	1	2	6	1	7	6	
RT191				RT191	**46**	4	1	6	1	1	10	1	
	RT194			RT194	**41**	1	1	9	9	1	3	2	
		RT196	1	RT196	New	1	1	2	2	1	5	3	New (ST144)
RT202				RT202	**20**	1	1	8	1	1	4	3	
	RT202			RT202	**24**	1	1	8	12	1	4	3	
		RT212	1	RT212	**5**	1	6	4	7	2	8	7	
	RT216	RT216	1	RT216	**33**	1	1	2	1	6	5	3	
RT220	RT220			RT220	**2**	1	1	2	1	5	3	1	
	RT224			RT224	**65**	1	1	7	3	1	5	6	
RT225	RT225			RT225	**12**	1	1	6	4	3	5	1	
	RT228			RT228	**92**	1	1	2	3	1	5	1	
		RT237	5	RT237	**11**	5	8	5	11	9	11	8	
		RT239	1	RT239	New	10	8	5	11	9	11	8	New (ST147)
		RT243	1	RT243	New	2	1	2	1	1	1	3	New (ST139)
	RT249			RT249	**51**	1	1	2	6	1	7	6	
		RT259	1	RT259	New	1	3	7	1	3	1	6	New (ST141)
	RT262	RT262	2	RT262	**3**	1	1	2	1	1	1	1	
		RT262	1	RT262	New	1	11	6	16	1	1	1	New (ST143)
		RT264	1	RT264	New	8	7	14	8	6	25	15	New (ST142)
		RT268	1	RT268	**3**	1	1	2	1	1	1	1	
		RT271	1	RT271	**6**	2	1	6	1	1	5	1	
		RT274	1	RT274	New	1	1	7	1	1	3	3	New (ST133)
		RT280	1	RT280	**11**	5	8	5	11	9	11	8	
		RT281	1	RT281	**11**	5	8	5	11	9	11	8	
		RT283	1	RT283	New	1	3	2	15	1	3	3	New (ST134)
		RT291	1	RT291	New	**5**	8	5	11	9	11	20	New (ST148)
	RT305			RT305	**3**	1	1	2	1	1	1	1	
	RT316			RT316	**59**	1	3	6	15	1	17	1	
	RT319			RT319	**74**	1	1	13	1	3	1	14	
	RT320			RT320	**46**	4	1	6	1	1	10	1	
	RT321			RT321	**41**	1	1	9	9	1	3	2	
	RT323			RT323	**31**	1	5	7	6	1	1	1	
	RT326			RT326	**91**	1	1	6	6	1	6	1	
	RT336			RT336	**60**	1	3	15	15	1	3	1	
					**30**	8	7	14	8	6	16	15	
					**47**	1	1	11	17	1	3	2	
					**61**	1	5	11	1	1	3	2	
					**64**	1	1	6	1	1	13	1	

**79**	1	3	2	1	1	1	16
**80**	1	1	13	1	3	1	3
**81**	3	1	3	8	6	9	11
**82**	1	1	6	1	1	1	3
**83**	1	1	6	1	1	3	1
**84**	1	9	2	1	1	1	1
**85**	1	10	7	1	1	3	3
**86**	3	7	3	8	6	19	11
**87**	3	7	3	8	6	20	10
**88**	9	7	3	8	6	18	10
**93**	3	7	3	8	11	20	10
**94**	2	1	6	1	1	10	1
**95**	1	5	11	17	1	3	1
**96**	1	6	4	7	12	8	7
**97**	1	1	11	1	1	22	1
**98**	1	1	2	6	1	1	3
**99**	1	11	6	6	1	12	12
**100**	1	1	6	19	2	24	1
**101**	1	2	2	1	1	23	1
**102**	1	1	2	1	5	5	1
**103**	1	1	2	15	8	5	3
**104**	1	1	13	1	1	1	6
**105**	2	2	4	1	1	1	3
**106**	2	1	2	10	1	5	1
**107**	4	1	6	1	3	1	1
**108**	1	1	6	1	8	5	5
**109**	3	12	10	18	6	18	15
**110**	1	1	2	1	13	3	1
**111**	1	1	2	1	1	3	3
**112**	1	1	7	3	1	5	3
**113**	2	1	17	1	1	5	1
**114**	1	1	9	9	1	1	18
**115**	1	1	2	6	1	7	3
**116**	1	1	9	9	1	22	5
**117**	1	1	7	1	2	5	1
**118**	1	13	2	1	1	1	19
**119**	1	1	2	20	1	5	1
**120**	1	1	1	1	5	3	1
**121**	3	7	3	8	7	2	10
**122**	1	1	4	1	1	1	10
**123**	1	14	9	1	1	3	2

Griffiths	Dingle	Stabler	Nos	Con	ST	adk	atpA	dxr	glyA	recA	sodA	tpi
RT027	RT027	RT027	53	RT027	**1**	1	1	1	10	1	3	5
RT036				RT036	**1**	1	1	1	10	1	3	5
		RT176	2	RT176	**1**	1	1	1	10	1	3	5
		RT002	1	RT002	**2**	1	1	2	1	5	3	1
RT014	RT014	RT014	2	RT014	**2**	1	1	2	1	5	3	1
RT020	RT020	RT020	2	RT020	**2**	1	1	2	1	5	3	1
RT076	RT076			RT076	**2**	1	1	2	1	5	3	1
RT220	RT220			RT220	**2**	1	1	2	1	5	3	1
RT001	RT001	RT001	15	RT001	**3**	1	1	2	1	1	1	1
RT009	RT009	RT009	1	RT009	**3**	1	1	2	1	1	1	1
RT072	RT072			RT072	**3**	1	1	2	1	1	1	1
RT115	RT115			RT115	**3**	1	1	2	1	1	1	1
	RT262	RT262	2	RT262	**3**	1	1	2	1	1	1	1
		RT268	1	RT268	**3**	1	1	2	1	1	1	1
	RT305			RT305	**3**	1	1	2	1	1	1	1
RT137	RT137			RT137	**4**	1	2	2	1	1	5	3
RT023	RT023	RT023	4	RT023	**5**	1	6	4	7	2	8	7
RT063				RT063	**5**	1	6	4	7	2	8	7
		RT212	1	RT212	**5**	1	6	4	7	2	8	7
RT005	RT005	RT005	2	RT005	**6**	2	1	6	1	1	5	1
		RT042	1	RT042	**6**	2	1	6	1	1	5	1
		RT050	1	RT050	**6**	2	1	6	1	1	5	1
		RT271	1	RT271	**6**	2	1	6	1	1	5	1
RT026	RT026	RT026	1	RT026	**7**	1	1	7	1	1	5	1
RT002	RT002	RT002	10	RT002	**8**	1	1	2	6	1	5	1
RT159				RT159	**8**	1	1	2	6	1	5	1
RT081	RT081	RT081	1	RT081	**9**	1	1	6	1	1	6	1
RT015	RT015	RT015	3	RT015	**10**	2	1	2	1	1	3	1
		RT116	1	RT116	**10**	2	1	2	1	1	3	1
		RT033	1	RT066	**11**	5	8	5	11	9	11	8
RT078	RT078	RT078	106	RT078	**11**	5	8	5	11	9	11	8
		RT126	10	RT126	**11**	5	8	5	11	9	11	8
		RT127	3	RT127	**11**	5	8	5	11	9	11	8
		RT237	5	RT237	**11**	5	8	5	11	9	11	8
		RT280	1	RT280	**11**	5	8	5	11	9	11	8
		RT281	1	RT281	**11**	5	8	5	11	9	11	8
RT003	RT003			RT003	**12**	1	1	6	4	3	5	1
		RT094	2	RT094	**12**	1	1	6	4	3	5	1
RT225	RT225			RT225	**12**	1	1	6	4	3	5	1
		RT014	1	RT014	**13**	1	1	6	1	5	3	1
RT129	RT129			RT129	**13**	1	1	6	1	5	3	1
RT014	RT014			RT014	**14**	1	1	2	1	5	5	3
RT010		RT010	3	RT010	**15**	1	1	6	1	8	5	1
	RT070			RT070	**15**	1	1	6	1	8	5	1
RT050	RT050	RT050	1	RT050	**16**	1	1	2	6	1	3	1
RT018	RT018	RT018	1	RT018	**17**	1	1	2	1	1	5	3
RT050	RT050	RT050	2	RT050	**18**	1	1	2	5	1	3	1
RT110	RT110			RT110	**19**	1	1	8	2	1	1	3
RT202				RT202	**20**	1	1	8	1	1	4	3

	RT097	RT097	1	RT097	**21**	2	2	2	1	1	1	3
RT023	RT023	RT023	1	RT023	**22**	1	1	4	7	2	8	7
	RT138			RT138	**23**	3	7	14	14	11	16	15
	RT202			RT202	**24**	1	1	8	12	1	4	3
	RT023			RT023	**25**	6	6	4	7	2	8	7
RT039				RT039	**26**	1	1	6	1	4	3	4
RT140	RT140	RT140	1	RT140	**26**	1	1	6	1	4	3	4
RT067				RT067	**27**	1	1	6	1	1	3	4
	RT020			RT020	**28**	1	1	2	1	1	3	1
		RT031	1	RT031	**29**	1	1	2	16	1	3	1
					**30**	8	7	14	8	6	16	15
	RT323			RT323	**31**	1	5	7	6	1	1	1
RT153				RT153	**32**	1	1	11	1	1	3	2
RT064		RT064	1	RT064	**33**	1	1	2	1	6	5	3
	RT216	RT216	1	RT216	**33**	1	1	2	1	6	5	3
RT056	RT056			RT056	**34**	1	5	7	1	1	3	1
		RT002	4	RT002	**35**	2	5	8	1	1	3	6
RT046	RT046	RT046	1	RT046	**35**	2	5	8	1	1	3	6
RT011	RT011			RT011	**36**	2	1	2	3	1	5	1
RT017	RT017	RT017	8	RT017	**37**	3	7	3	8	6	9	11
RT060				RT060	**38**	3	7	10	8	6	2	9
RT085		RT085	1	RT085	**39**	3	7	10	8	7	2	10
RT035				RT035	**40**	4	5	6	1	3	1	1
	RT106			RT106	**41**	1	1	9	9	1	3	2
		RT135	1	RT135	**41**	1	1	9	9	1	3	2
	RT194			RT194	**41**	1	1	9	9	1	3	2
	RT321			RT321	**41**	1	1	9	9	1	3	2
RT106	RT106	RT106	9	RT106	**42**	1	1	2	1	1	7	1
RT118		RT118	1	RT118	**42**	1	1	2	1	1	7	1
RT174	RT174			RT174	**42**	1	1	2	1	1	7	1
	RT054	RT054	2	RT054	**43**	1	7	6	1	1	5	6
	RT015	RT015	4	RT015	**44**	2	5	2	1	1	3	1
		RT062	1	RT062	**44**	2	5	2	1	1	3	1
	RT013	RT013	1	RT013	**45**	4	1	6	1	1	5	1
		RT087	2	RT087	**46**	4	1	6	1	1	10	1
RT191				RT191	**46**	4	1	6	1	1	10	1
	RT320			RT320	**46**	4	1	6	1	1	10	1
					**47**	1	1	11	17	1	3	2
		RT002	1	RT002	**48**	1	1	2	1	1	5	1
		RT030	1	RT030	**48**	1	1	2	1	1	5	1
	RT038			RT038	**48**	1	1	2	1	1	5	1
	RT104			RT104	**48**	1	1	2	1	1	5	1
	RT014	RT014	1	RT014	**49**	1	1	2	1	5	3	3
	RT014			RT014	**50**	1	1	6	1	5	3	17
RT186	RT186	RT186	1	RT186	**51**	1	1	2	6	1	7	6
	RT249			RT249	**51**	1	1	2	6	1	7	6
	RT139	RT139	1	RT139	**52**	1	1	2	6	1	12	1
		RT059	1	RT059	**53**	1	2	2	1	1	5	1
RT103	RT103			RT103	**53**	1	2	2	1	1	5	1
RT012	RT012	RT012	3	RT012	**54**	1	4	7	1	1	3	3

	RT070	RT070	1	RT070	**55**	1	1	6	6	1	12	12
RT021	RT021	RT021	1	RT021	**56**	1	3	6	3	1	5	1
	RT003	RT003	1	RT003	**57**	1	1	6	4	3	5	13
	RT056			RT056	**58**	1	5	7	1	1	13	1
	RT316			RT316	**59**	1	3	6	15	1	17	1
	RT336			RT336	**60**	1	3	15	15	1	3	1
					**61**	1	5	11	1	1	3	2
		RT036	1	RT036	**62**	1	1	1	9	1	3	1
	RT053	RT053	1	RT053	**63**	1	1	7	5	1	3	3
					**64**	1	1	6	1	1	13	1
	RT224			RT224	**65**	1	1	7	3	1	5	6
	RT022	RT022	1	RT022	**66**	1	1	2	6	1	5	3
RT019	RT019			RT019	**67**	1	1	9	9	1	3	5
	RT020			RT020	**68**	1	4	2	1	5	3	1
RT072				RT072	**69**	1	1	7	1	1	1	1
	RT021			RT021	**70**	1	3	6	3	1	14	1
	RT013			RT013	**71**	4	1	6	1	1	15	1
	RT005			RT005	**72**	2	1	6	13	1	5	1
	RT103			RT103	**73**	1	2	12	1	1	5	1
	RT319			RT319	**74**	1	1	13	1	3	1	14
	RT062			RT062	**75**	7	3	13	1	10	1	1
	RT103			RT103	**76**	1	1	6	1	1	1	1
	RT011			RT011	**77**	2	1	6	1	3	5	1
	RT013			RT013	**78**	4	1	6	1	1	1	1
					**79**	1	3	2	1	1	1	16
					**80**	1	1	13	1	3	1	3
					**81**	3	1	3	8	6	9	11
					**82**	1	1	6	1	1	1	3
					**83**	1	1	6	1	1	3	1
					**84**	1	9	2	1	1	1	1
					**85**	1	10	7	1	1	3	3
		RT017	1	RT017	**86**	3	7	3	8	6	19	11
					**86**	3	7	3	8	6	19	11
					**87**	3	7	3	8	6	20	10
					**88**	9	7	3	8	6	18	10
	RT005			RT005	**89**	2	1	6	1	1	21	1
	RT005			RT005	**90**	2	1	16	1	1	5	1
	RT326			RT326	**91**	1	1	6	6	1	6	1
	RT228			RT228	**92**	1	1	2	3	1	5	1
					**93**	3	7	3	8	11	20	10
					**94**	2	1	6	1	1	10	1
					**95**	1	5	11	17	1	3	1
					**96**	1	6	4	7	12	8	7
					**97**	1	1	11	1	1	22	1
					**98**	1	1	2	6	1	1	3
					**99**	1	11	6	6	1	12	12
					**100**	1	1	6	19	2	24	1
					**101**	1	2	2	1	1	23	1
					**102**	1	1	2	1	5	5	1
					**103**	1	1	2	15	8	5	3

			**104**	1	1	13	1	1	1	6	
			**105**	2	2	4	1	1	1	3	
			**106**	2	1	2	10	1	5	1	
			**107**	4	1	6	1	3	1	1	
			**108**	1	1	6	1	8	5	5	
			**109**	3	12	10	18	6	18	15	
			**110**	1	1	2	1	13	3	1	
			**111**	1	1	2	1	1	3	3	
			**112**	1	1	7	3	1	5	3	
			**113**	2	1	17	1	1	5	1	
			**114**	1	1	9	9	1	1	18	
			**115**	1	1	2	6	1	7	3	
			**116**	1	1	9	9	1	22	5	
			**117**	1	1	7	1	2	5	1	
			**118**	1	13	2	1	1	1	19	
			**119**	1	1	2	20	1	5	1	
			**120**	1	1	1	1	5	3	1	
			**121**	3	7	3	8	7	2	10	
			**122**	1	1	4	1	1	1	10	
			**123**	1	14	9	1	1	3	2	
RT002	1	RT002	New	1	1	2	6	1	5	4	New (ST146)
RT005	1	RT005	New	2	1	6	8	1	5	1	New (ST131)
RT011	1	RT011	New	2	1	2	16	1	5	1	New (ST138)
RT014	1	RT014	New	**5**	**1**	6	1	5	3	1	New (ST132)
RT029	1	RT029	New	1	1	2	3	1	3	1	New (ST137)
RT052	1	RT052	New	2	1	6	16	1	5	13	New (ST136)
RT081	1	RT081	New	2	1	2	1	1	1	3	New (ST139)
RT087	1	RT087	New	**4**	1	6	1	1	10	12	New (ST145)
RT106	1	RT106	New	1	1	2	8	1	7	1	New (ST135)
RT107	1	RT107	New	2	1	2	1	1	1	3	New (ST139)
RT111	1	RT111	New	1	1	9	9	1	1	2	New (ST140)
RT196	1	RT196	New	1	1	2	2	1	5	3	New (ST144)
RT239	1	RT239	New	10	8	5	11	9	11	8	New (ST147)
RT243	1	RT243	New	2	1	2	1	1	1	3	New (ST139)
RT259	1	RT259	New	1	3	7	1	3	1	6	New (ST141)
RT262	1	RT262	New	1	11	6	16	1	1	1	New (ST143)
RT264	1	RT264	New	8	7	14	8	6	25	15	New (ST142)
RT274	1	RT274	New	1	1	7	1	1	3	3	New (ST133)
RT283	1	RT283	New	1	3	2	15	1	3	3	New (ST134)
RT291	1	RT291	New	**5**	8	5	11	9	11	20	New (ST148)
