# Supplementary material for: Macro and Micro Diversity of Clostridium difficile Isolates from Diverse Sources and Geographical Locations
Source: PLoS One. 2012 Mar 2;7(3):e31559. doi: 10.1371/journal.pone.0031559 (PMC3292544; doi:10.1371/journal.pone.0031559)
Supplement: Data S5 — Mini clusters within clade 1. Closely related sequence types cluster in to mini clades within the larger heterogenous clade 1. MC = micro cluster identifier, adk, atpA, dxr, glyA, recA, sodA and tpi columns indicate allele numbers (red = consensus, ‘.’ = same as consensus), SNP column indicates the number of single nucleotide polymorphisms between consensus allele and deviant allele (1Δ indicates a single base pair deletion within sodA allele 21), associated RTs column indicates any known PCR ribotypes associated with this ST. (PDF) [file pone.0031559.s013.pdf]

MC	ST	adk	SNP	atpA	SNP	dxr	SNP	glyA	SNP	recA	SNP	sodA	SNP	tpi	SNP	Associated RTs
	18	.		.		.		5	1	.		.		.		RT050
	8	.		.		.		.		.		5	1	.		RT002, RT159
	16	1		1		2		6		1		3		1		RT050
	31	.		5	1	7	1	.		.		1	1	.		RT323
	51	.		.		.		.		.		7	3	6	2	RT186, RT249
	52	.		.		.		.		.		12	2	.		RT139
A	91	.		.		6	1	.		.		6	2	.		RT326
	S10.1920	.		.		.		.		.		5	1	4	1	RT002
	55	.		.		6	1	.		.		12	2	12	1	RT070
	99	.		11	1	6	1	.		.		12	2	12	1	
	66	.		.		.		.		.		5	1	3	1	RT022
	98	.		.		.		.		.		1	1	3	1	
	115	.		.		.		.		.		7	3	3	1	
	10	2		1		2		1		1		3		1		RT015, RT116
B	35	.		5	1	8	1	.		.		.		6	2	RT046
	44	.		5	1	.		.		.		.		.		RT015, RT062
C	12	1		1		6		4		3		5		1		RT003, RT094
	57	.		.		.		.		.		.		13	1	RT003
	13	1		1		6		1		5		3		1		RT014, RT129
D	50	.		.		.		.		.		.		17	1	RT014
	Ai18	5	5	.		.		.		.		.		.		RT014
E	14	1		1		2		1		5		5		3		RT014
	49	.		.		.		.		.		3	1	.		RT014
F	15	1		1		6		1		8		5		1		RT010, RT070
	108	.		.		.		.		.		.		5	1	
	19	1		1		8		2		1		1		3		RT110
G	20	.		.		.		1	1	.		4	2	.		RT202
	24	.		.		.		12	1	.		4	2	.		RT202

H	26	1		1		6		1		4		3		4		RT140
	27	.		.		.		.		1	1	.		.		RT067
	29	.		.		.		16	1	.		3	1	.		RT031
	92	1		1		2		3		1		5		1		RT228
	CD1171	.		.		.		.		.		3	1	.		RT029
	CD1080	2	1	.		6	1	16	1	.		.		13	1	RT052
	36	2	1	.		.		.		.		.		.		RT011
	CD1214	2	1	.		.		16	1	.		.		.		RT011
I	56	.		3	1	6	1	.		.		.		.		RT021
	70	.		3	1	6	1	.		.		14	3	.		RT021
	59	.		3	1	6	1	15	2	.		17	3	.		RT316
	100	.		.		6	1	19	2	2	1	24	3	.		
	CD959	.		11	1	6	1	16	1	.		1	2	.		RT262
	60	.		3	1	15	1	15	2	.		3	1	.		RT336
	103	.		.		.		15	2	8	1	.		3	1	
	CD1061	.		3	1	.		15	2	.		3	1	3	1	RT283
	78	4		1		6		1		1		1		1		RT013
	40	.		5	1	.		.		3	1	.		.		RT035
	107	.		.		.		.		3	1	.		.		
K	45	.		.		.		.		.		5	3	.		RT013
	71	.		.		.		.		.		15	4	.		RT013
	46	4		1		6		1		1		10		1		RT087, RT191, RT320
	RPH35	.		.		.		.		.		.		12	1	RT087
	42	1		1		2		1		1		7		1		RT118
L	42															RT174
	CD1077	.		.		.		8	2	.		.		.		RT106
	112	.		.		.		3	1	.		5	1	.		
	63	.		.		.		5	1	.		.		.		RT053
M	CD1010	1		1		7		1		1		3		3		RT274
	54	.		4	2	.		.		.		.		.		RT012
	85	.		10	3	.		.		.		.		.		
N	74	1		1		13		1		3		1		14		RT319
	80	.		.		.		.		.		.		3	1	

	79	1	3		2		1	1		1	16		
O	104	.	1	1	13	1	.	.		.	6	1	
	CD871	.	.		7	1	.	3	1	.	6	1	RT259
